# Application of Magnetic Resonance to Assess Lyophilized Drug Product Reconstitution

**DOI:** 10.1007/s11095-019-2591-x

**Published:** 2019-03-22

**Authors:** Thomas A. Partridge, Mahammad Ahmed, Sureshkumar B. Choudhary, Christopher F. van der Walle, Sajal M. Patel, Steven M. Bishop, Mick D. Mantle

**Affiliations:** 1 Department of Chemical Engineering and Biotechnology, University of Cambridge, Philippa Fawcett Drive, Cambridge, CB3 0AS UK; 2 Conjugation Group, Spirogen Ltd, QMB Innovation Centre, 42 New Road, London, E1 2AX 20878 UK; 3 Dosage Form Design and Development, AstraZeneca, One Medimmune Way, Gaithersburg, MD 20878 USA; 4 Dosage Form Design and Development, Spirogen Ltd, Sir Aaron Klug Building, Granta Park, Cambridge, CB21 6GH UK

**Keywords:** dissolution, lyophilization, magnetic resonance imaging, protein concentration, protein formulation, reconstitution, T_2_ relaxation

## Abstract

**Purpose:**

Dynamic *in-situ* proton (^1^H) magnetic resonance imaging (MRI) and ^1^H T_2_-relaxometry experiments are described in an attempt to: (i) understand the physical processes, that occur during the reconstitution of lyophilized bovine serum albumin (BSA) and monoclonal antibody (mAb) proteins; and (ii) objectify the reconstitution time.

**Methods:**

Rapid two-dimensional ^1^H MRI and diffusion weighted MRI were used to study the temporal changes in solids dissolution and characterise water mass transport characteristics. One-shot T_2_ relaxation time measurements were also acquired in an attempt to quantify the reconstitution time. Both MRI data and T_2_-relaxation data were compared to standard visual observations currently adopted by industry. The ^1^H images were further referenced to MRI calibration data to give quantitative values of protein concentration and, percentage of remaining undissolved solids.

**Results:**

An algorithmic analysis of the ^1^H T_2_-relaxation data shows it is possible to classify the reconstitution event into three regimes (*undissolved*, *transitional* and *dissolved*). Moreover, a combined analysis of the 2D ^1^H MRI and ^1^H T_2_-relaxation data gives a unique time point that characterises the onset of a reconstituted protein solution within well-defined error bars. These values compared favourably with those from visual observations. Diffusion weighted MRI showed that low concentration BSA and mAb samples showed distinct liquid-liquid phase separation attributed to two liquid layers with significant density differences.

**Conclusions:**

T_2_ relaxation time distributions (whose interpretation is validated from the 2D ^1^H MR images) provides a quick and effective framework to build objective, quantitative descriptors of the reconstitution process that facilitate the interpretation of subjective visual observations currently adopted as the standard practice industry.

**Electronic supplementary material:**

The online version of this article (10.1007/s11095-019-2591-x) contains supplementary material, which is available to authorized users.

## Introduction

Lyophilization is a unit operation used extensively in the biopharmaceutical industry aimed at producing dry stable protein formulations containing 0.1–4% water (with <0.3% often achieved in modern formulation), thereby increasing the stability and shelf life of the product (1–5). Prior to patient administration, the lyophilized drug product must be reconstituted. The reconstitution times of such formulations can vary significantly, from minutes to hours with the longer reconstitution times generally observed for high concentration protein formulations (6–9). Lyophilization is relatively complicated and involves a number of variables, which have long been considered in the literature using a wealth of analytical techniques, both from a process performance and product quality point of view (8–10). However, factors influencing the reconstitution of lyophilized drug products are still poorly understood, with the effect of even common steps of the lyophilization process still debated. For example, annealing, which is often included during freezing to improve the crystallisation and pore size, has been shown to both increase (11) and decrease (12,13) reconstitution times.

Reconstitution procedures can vary from one therapeutic protein product to another, making empirical observations of reconstitution times subjective and difficult to compare. Recently, efforts have been made to standardise definitions such that the reconstitution time is generally recorded after injection ends until all visible solids have dissolved (14,15), while amounts of diluent and agitation methods are typically stated. The United States Pharmacopeia (USP) Pharmacopeial Forum (16) indicates that to ensure full reconstitution there should be no “visible residue” and that “the constituted solution is not significantly less clear than an equal volume of the diluent”. It has also given revisions (17) on guidelines of visual inspection largely based on the work of Melchore and Berdovich (18). It is recognised by USP that this is a probabilistic determination and so remains subjective in nature and dependent on scenario. Werk *et al*. (19) have attempted to create an automated method to determine the endpoint of reconstitution using an impedance based method, which was shown to be successful so long as temperature and agitation were controlled. Other techniques used UV (20) and a laser particle sizer (21) to determine reconstitution endpoint but were not performed *in situ*.

Large variations have been observed in reconstitution times for different products with values as long as 90 min seen in some snake anti-venoms (22) and anywhere from a few minutes (or less) (23) to 40 min (15) in the case of some monoclonal antibodies. A vast number of product parameters along with analytical techniques have been investigated and applied to studying reconstitution after it has occurred and include particle size distribution and porosity measurements (15,23–28), lyophilization cooling profile and annealing (11–13,15,29), diluent volume and vial size (23,29,30), additives and wetting agents (23,31,32), protein product structure (23,33,34), reconstitution under vacuum (23) and agitation (15,23,29). However, to the best of our knowledge there have been no analytical experimental studies that have been applied non-invasively to study the real-time reconstitution process of a lyophilized product beyond determination of reconstitution endpoint (19). Two-dimensional (2D) ^1^H magnetic resonance (MR) measurements have, however, been more widely used to study protein stability and mobility using relaxation parameters, such as to investigate miscibility with changing formulations (35), determining sugar types and content (36), investigating lyophilized systems stored at different relative humidity (37), and the effect of different polymer excipients on formulation (38). MR imaging has also been applied to look at the end point of lyophilization in potatoes (39). T_2_ relaxation NMR has recently been used to investigate protein aggregation using a low field bench top NMR spectrometer. The results were shown to be superior to traditional methods such as size exclusion chromatography, dynamic light scattering and micro-flow imaging (40). Solid state NMR measurements on lyophilized drug products using T_1_ and T_1ρ_ relaxometry are more common, for example looking at annealing (41) and protein stability (42,43).

In this paper we report the use of rapid single-shot one-dimensional (1D) ^1^H nuclear magnetic resonance (NMR) T_2_–relaxation time measurements combined with fast 2D ^1^H magnetic resonance imaging (MRI) methods as a non-invasive tool for monitoring the reconstitution process within a standard lyophilized sample vial. Single shot magnetic resonance T_2_-relaxation data (analysed using numerical inversion) coupled with spatially resolved MRI data allow us to objectively partition the in-situ reconstitution process into three distinct regimes denoted: (0) undissolved; (1) transitional; (2) dissolved. The analysis provides two distinct time points associated with the onset of steady state morphological behaviour and a time point where reconstitution can be considered complete within error limits. The MR image data can be calibrated to external reference samples to provide measures of protein concentrations and remaining undissolved solids content after reconstitution for comparison with standard UV analysis. The combination of magnetic resonance imaging and relaxation methods are then used to provide a physical interpretation of the reconstitution process.

## Experimental

### Materials and Lyophilization Methods

Bovine Serum Albumin (BSA) was purchased as dry powder (product code A4503, Lot #SLBP3628V, MW 66.4 kDa, pI ~4.8, extinction coefficient 0.67 (mg/ml)/cm from Sigma-Aldrich (Merck), UK. Samples of a recombinant, human IgG1 monoclonal antibody (hereafter referred to as ‘mAb’), MW 143 kDa, pI ~9, extinction coefficient 1.41 (mg/ml)/cm, were prepared by AstraZeneca plc, Cambridge, UK, in 13 mm Schott Type I clear tubular 3 ml glass vials (West Pharmaceutical Services) and stoppered with Daikyo D777–1 13 mm single vent lyo-stoppers (West Pharmaceutical Services). A VirTis Genesis freeze drier was used with an annealing step included in the cooling profile. After this the pressure was reduced to 100 mTorr and the temperature lowered to −25°C for 55 h for primary drying. The temperature was then ramped at 0.1 °C per minute to 40 °C while maintaining 100 mTorr pressure for 6 h to allow for secondary drying. BSA was dissolved at a target concentration of 150 mg/ml in 240 mM sucrose, 20 mM histidine/histidine HCl, 0.02% polysorbate 80 (*w*/*v*) pH 6.0 (‘BSA buffer’), and diluted to 20 mg/ml over a range of concentrations. The mAb was first dialysed (20 kDa MWCO Slide-A-Lyzer™ Dialysis Cassettes, Thermo Fisher Scientific, UK, following the manufacturer’s instructions) into 80 mM arginine HCl, 120 mM sucrose, 20 mM histidine/histidine HCl, 0.02% polysorbate 80 (*w*/*v*) pH 6.0 (‘mAb buffer’), concentrated to around 150 mg/ml (30 kDa MWCO Amicon Ultra centrifugal filters, Merck, UK) and diluted to 20 mg/ml over a range of concentrations. The concentration of representative samples for each dilution step was measured in triplicate by UV-absorbance at 280 nm (A280) using a Trinean DropSense Multi-Channel Spectrophotometer (Unchained Labs Inc.). The effect of formulation and lyophilization process parameters are outside the scope of this work. For brevity samples will be referred to by their protein concentration only e.g. BSA with a target protein concentration of 20 mg/ml will be referred to as BSA 20.

**Fig. 1:**
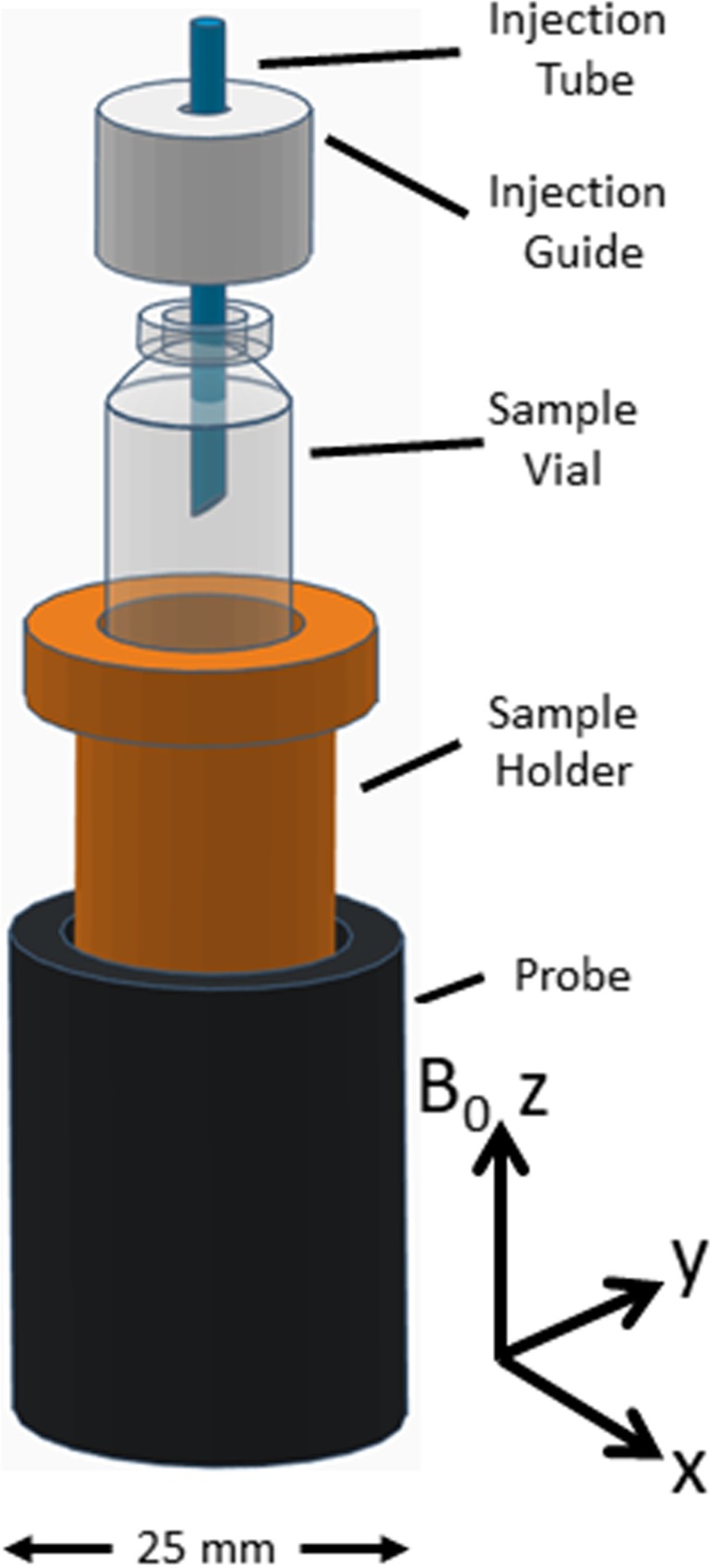
Schematic of the *in situ* injection setup.

The *in situ* sample vial holder for use within the magnet was made of Poly-Ether Ether Ketone and the setup driven by a computer controlled Harvard Instruments Model 22 syringe pump. A reconstitution volume of 1 ml of 15 MΩcm deionised water was used for all samples bringing them back to their original concentrations. A flow rate of 5 ml/min was used (i.e. injection duration was 12 s). A schematic of the sample vial holder is shown in Fig. 1.

#### Magnetic Resonance Methods and Data Analysis Procedures

All magnetic resonance experiments were conducted on a Bruker Biospin Ultrashield 9.4 T (400 MHz ^1^H frequency) widebore magnet combined with a Bruker Biospin Avance spectrometer, using a 25 mm micro-imaging probe. One shot T_2_ data were acquired using a standard Carr-Purcell-Meiboom Gill (CPMG), pulse sequence; the interecho spacing was 5 ms and a total of 512 echoes were acquired. The oneshot T_2_ data were acquired every 12.2 s up to a total time of t = 1565 s and 3620 s for BSA and mAb samples respectively. The data was analysed using a one dimensional numerical inversion, sometime referred to as an Inverse Laplace Transform (ILT), as described in the literature (44–52). The Laplace inversion was adopted here because it is a general and robust method that is used when no prior information about the number and distribution of T_2_ values is known. At the end of each experiment the sample was removed from the magnet, swirled gently for 1 min by hand and then replaced back into the magnet and three further one shot experiments were acquired and subsequently transformed using numerical inversion to obtain a T_2_ data set representative of time t = ∞ (t_inf_). Three repeats (*n* = 3) were performed for all samples.

#### T_2_ Partitioning

**Fig. 2:**
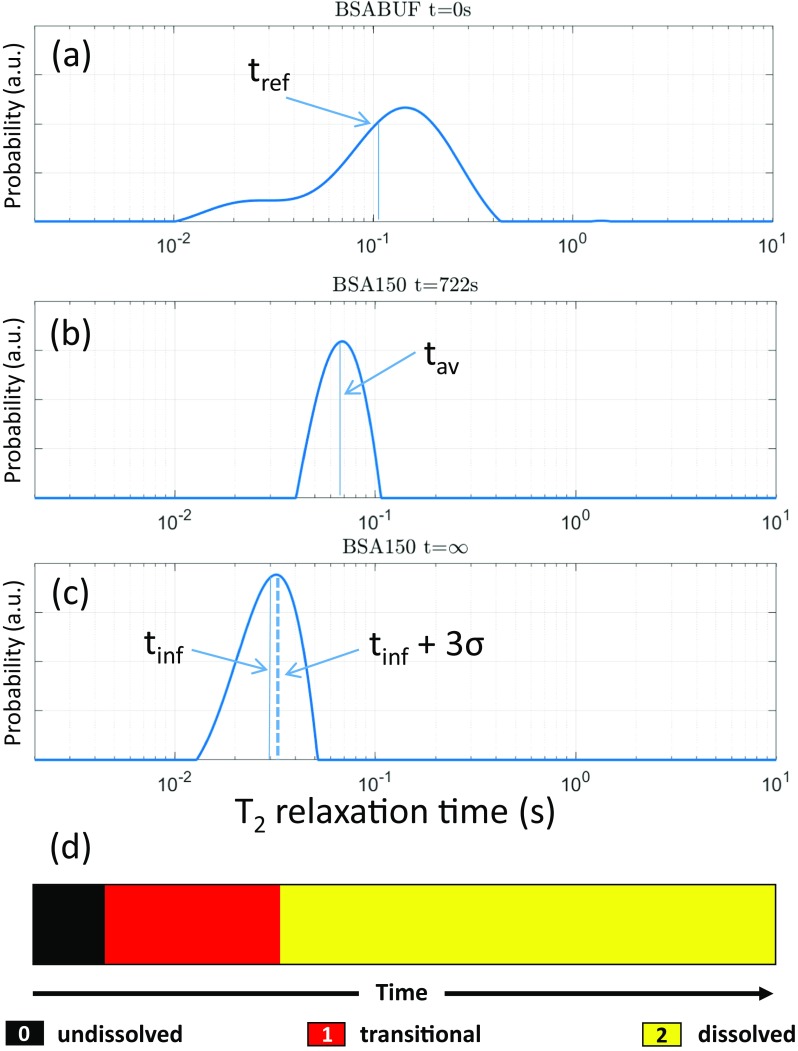
(**a**) T_2_ distribution obtained at time zero for the BSA buffer solution. Solid vertical line represents t_ref_, the weighted average T_2_ value for BSA buffer at t = 0 s. (**b**) T_2_ distribution for BSA 150 taken at t = 722 s, the solid vertical line represents t_av_, the weighted averaged T_2_ value. (**c**) T_2_ distribution from BSA 150 extracted after swirling the sample. Solid line represents the weighted average T_2_ value at t = ∞ (t_inf_). Dashed line represents t_inf_ + 3σ. (**d**) Schematic 1D color map that evolves with time from the assignment of the 3 integer values following the partitioning criteria (i-vi) listed in the “T_2_ Partitioning” section. Note the sharp transitions from black to red and red to yellow represent an average weighted T_2_ value from three independent samples (*n* = 3).

Following numerical inversion of each *in situ* one-shot T_2_ experiment the resulting relaxation time distributions are partitioned according to the following procedure:

(i) A weighted average ‘reference’ T_2_ value (t_ref_), of a pure buffer solution, i.e. without BSA or mAb, is calculated for the first time point of the reconstitution process at t = 0 s (Fig. 2(a)). The weighted average of a T_2_ distribution is calculated by multiplying each fractional probability value by its corresponding T_2_ relaxation time and then summing those values. This sum is then divided by the sum of the fractional probabilities to give the weighted average.
(ii) A weighted average T_2_ value (t_av_) of each individual T_2_ distribution for BSA and mAb samples is calculated for all individual time points during the reconstitution (Fig. 2(b)).
(iii) A weighted average T_2_ value of swirled samples, to give a single T_2_ relaxation value at t = ∞, is calculated and denoted t_inf_ (Fig. 2(c)).
(iv) If the weighted average, t_av_ is greater than t_ref_, then those values are assigned an arbitrary value of 0 and correspond to samples with undissolved large bulk solids; time points where this condition is true are subsequently referred to as “*undissolved*”.
(v) If t_inf_ + 3σ < t_av_ < t_ref_, then the T_2_ values are given an arbitrary value of 1 and are representative of a system that is “*transitional*” (see Fig. 2(b)).
(vi) The remaining T_2_ values i.e. those with t_av_ < t_inf_ + 3σ are considered to represent a system that has fully dissolved, and are assigned an arbitrary value of 2 (see Fig. 2(c,d)) and denoted “*dissolved*”

This procedure when applied to the weighted T_2_ relaxation distributions, results in 1D color maps of a particular sample with three values representing undissolved (0 = black), transitional (1 = red), and dissolved (2 = yellow) during the time evolution of the reconstitution process. An example of this T_2_ partitioning is shown in Fig. 2(d). Error bars in the T_2_ relaxation data are calculated from three separate reconstitution experiments. We note that the width of the T_2_ distribution arises from the regularisation procedure that is used in conjunction with the numerical inversion (44–54).

#### MR Imaging

For 2D ^1^H MRI measurements, a single shot Rapid Acquisition with Relaxation Enhancement (RARE) sequence was used for reconstitution experiments with a rare factor of 128 and an inter-echo echo time of 2.5 ms giving an effective T_2_ weighting of the ^1^H signal from water of 160 ms. A recycle time of 8 s was used for all imaging to allow >95% (~3 × T_1_) of the ^1^H signal from water to relax back to equilibrium following excitation. 2D images in the ZX plane were recorded using 128 (Z) × 64 (X) pixels with a spatial resolution of 125 × 250 μm. The slice thickness was 16 mm meaning the images are a projection of the entire y-axis onto a 2D plane. In order to capture the dynamics of the reconstitution process 2D ^1^H RARE images were acquired every 8 s so that T_1_-relaxation contrast was negligible and only T_2_-contrast needed to be accounted for. Up to 200 ^1^H 2D RARE images were acquired in a typical reconstitution run resulting in a total imaging time of 26 min. After 26 min, the sample was physically removed from the magnet, swirled for 2 min by hand, and then placed back in the magnet for six final shot XY and ZX RARE images giving an image at t = ∞. For mAbs, 440 ^1^H 2D RARE images were recorded (~1 h), before the sample was removed and swirled, while mAb 125 and 150 samples had to be set aside for longer to allow full reconstitution. Three repeat experiments were performed for both BSA and mAb. All MR images were processed off-line using Matlab™.

#### Diffusion Weighted Imaging

A pulse gradient spin echo (PGSE) diffusion weighted imaging RARE sequence (55) was used to obtain spatially resolved maps of the molecular self-diffusion coefficient of the water in certain samples. All the ^1^H RARE imaging parameters in the PGSE weighted RARE sequence were identical to those used for standard RARE imaging described above. The PGSE parameters were as follows; 8 increments of the diffusion gradient, g, ranging from 0.1 T m^−1^ to 1.0 T m^−1^, δ = 3.0 ms, Δ = 8.0 ms, number of scans = 4. Each pixel in the series of eight diffusion weighted images were fitted to the standard Stejskal-Tanner equation in Matlab with a single fitting parameter giving the self-diffusion coefficient *D* of the water in m^2^ s^−1^.

#### Protein Concentration Calibration Procedure Using MRI

In order to convert the t = ∞ ^1^H intensity images into protein concentration images, a calibration procedure was developed and is briefly described below with additional material in the supplementary information S1-S3.

1. Following *in situ* measurement, the vial is removed from the magnet and swirled until all solids were dissolved.
2. Serial dilutions were prepared at six concentrations from the swirled vials, starting with no dilution.
3. The samples were then transferred from the reconstituted vials into standard 5 mm NMR tubes
4. Up to seven 5 mm NMR tubes were placed in a holder (6 sample concentrations and water) and then into the MRI magnet. A single shot 1 mm slice selective RARE XY image was taken with the same echo-time (TE = 2.5 ms) and RARE factor (128) as that used for the ZX reconstitution imaging. This procedure ensures exactly the same T_2_ weighting (contrast) is applied to the calibration phantom of 5 mm NMR tubes to that of the vials during the actual reconstitution experiments. Example images for all BSA and mAb samples can be seen in Figure S1.
5. Following Fourier transformation of the raw calibration image data, a 3 pixel grid of points was taken from the centre of each individual 5 mm phantom and averaged to give a single value at each individual serial dilution. The data from such analysis are plotted in Figure S2 and show an exponential decrease in ^1^H signal intensity for all phantoms and serial dilutions.
6. Three fitting parameters are determined for each individual BSA and mAb concentration. Each fitting parameter is then individually plotted against the protein concentration determined by UV analysis thus yielding a general formula or master curve for each fit parameter. This general formula can then be used to calculate the “NMR” reconstitution protein concentration value, Figure S3.
7. Protein concentration images/maps are corrected for the cylindrical projection geometry by dividing each map by one obtained from pure water.

## Results & Discussion

### Lyophilized Product Appearance

**Fig. 3:**
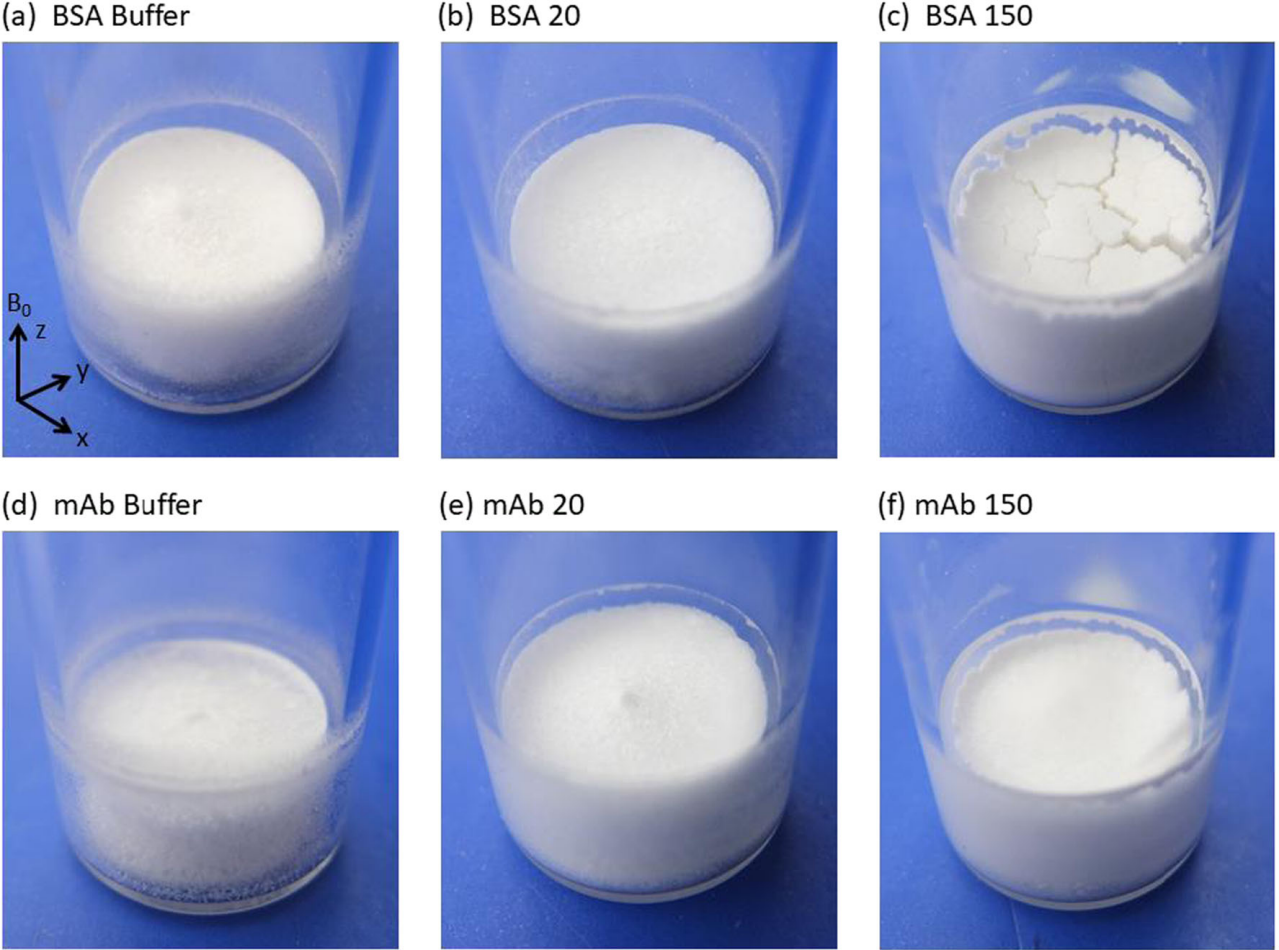
Appearance of lyophilized: (**a**) BSA buffer, (**b**) BSA 20, (**c**) BSA 150, (**d**) mAb buffer, (**e**) mAb 20, and (**f**) mAb 150. See Table I for actual protein concentrations. Z axis is in the direction of the vial vertical.

Figure 3 shows the appearance of lyophilized BSA and mAb samples, as well as their respective lyophilized buffers. Product shrinkage increases with protein concentration for both BSA and mAb, with cracking observed in BSA 125 and BSA 150, Fig. 3(c), and very minor cracking in the mAb 150 preparation, Fig. 3(f). In addition, Fig. 3 also shows solids attached to the inner rim of the vial for all samples.

#### Protein Concentrations and Visual Reconstitution Times

To ensure comparability of visual data available in the supplementary information S5, S7 with that of MRI data, no agitation of any sort was applied. Reconstitution times based on visual observation are summarized in Table I. The reconstitution time was taken from the end of injection until no visible solid particulate matter was observable. This is noted to cause a considerable sample to sample variation at all concentrations due to persistent solid particles in some instances. In the case of higher concentration mAb samples (≥100 mg/ml) the times are inflated by tiny solid residue adhered to the inner vertical walls of the glass vials. The reconstitution times for both BSA and mAb samples were generally found to increase with protein concentration, with much longer reconstitution times evident for higher concentrations (≥100 mg/ml), particularly in the case of the mAb samples.

**Table I:** Measured Weighted Mean Protein Concentration Determined by UV Absorbance and Visual Reconstitution Times for n Sample Preparations

Formulation	Weighted Mean Concentration ± SD (mg/ml)	*n*	Reconstitution Time ± SD (s)	*n*
BSA buffer	**–**	–	70 ± 20	3
BSA 20	20 ± 2	11	90 ± 20	3
BSA 50	51 ± 2	8	140 ± 50	3
BSA 75	79 ± 3	12	220 ± 60	3
BSA 100	104 ± 4	8	260 ± 40	3
BSA 125	124 ± 3	11	1300 ± 100	3
BSA 150	159 ± 8	10	2100 ± 100	3
mAb buffer	**–**	–	40 ± 20	3
mAb 20	19 ± 2	10	130 ± 30	3
mAb 50	49 ± 2	8	260 ± 40	3
mAb 75	75 ± 1	10	600 ± 100	3
mAb 100	105 ± 1	8	3000 ± 900*	3
mAb 125	128 ± 9	10	6500 ± 400*	3
mAb 150	153 ± 4	10	10,000 ± 1000*	3

*mAb reconstitution times significantly longer due to product sticking to the inner rim of the glass vials

### Magnetic Resonance Studies of the Reconstitution Process

The following sections first focus on the interpretation of the *in situ* 2D ^1^H MRI data; this is followed by a description and discussion of the T_2_ relaxation distributions and their subsequent partitioning analysis. Finally, a discussion combining both imaging and relaxation data is presented to understand the physical interpretation of the T_2_ relaxation time distributions.

#### MRI of Lyophilized Product Reconstitution

##### BSA and mAb 20 mg/ml Samples

**Fig 4:**
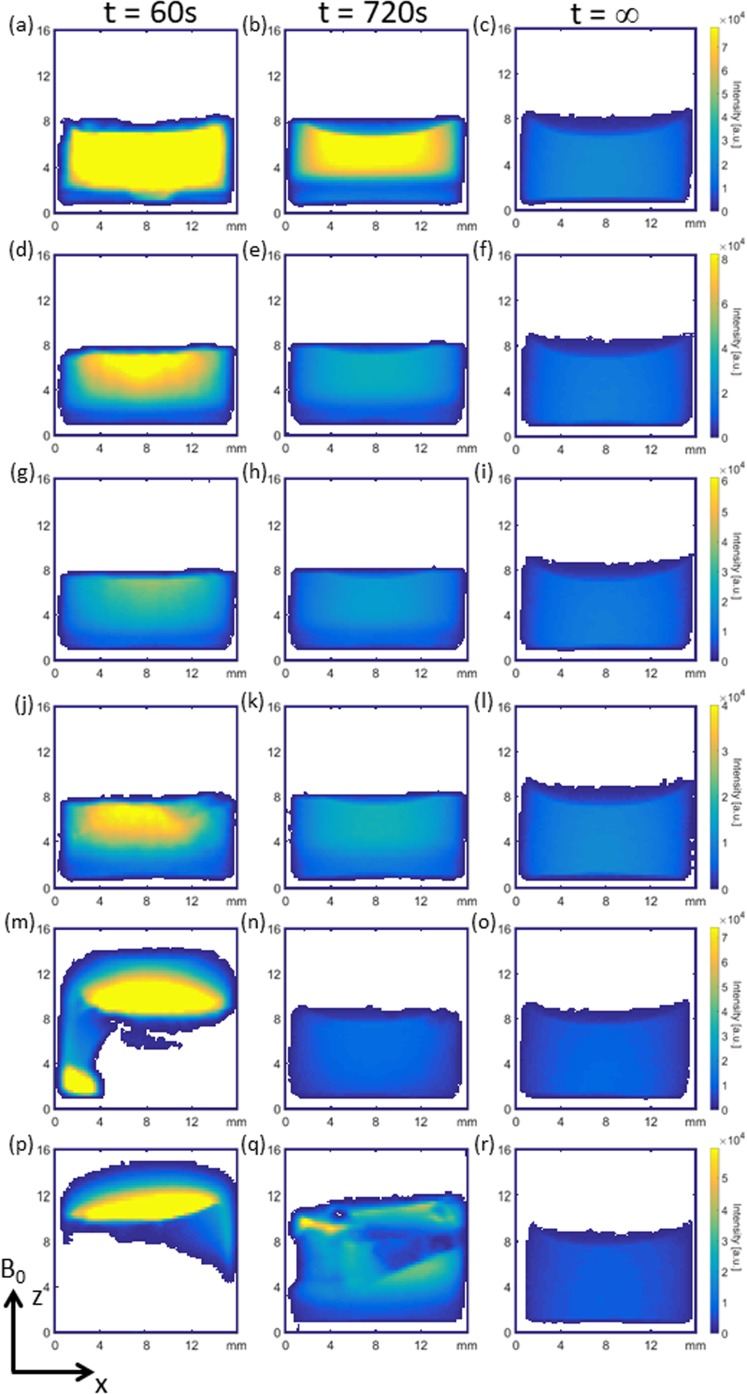
^1^H MRI images at t = 60 s, 720 s and post swirling (t = ∞) respectively for: (**a**) - (**c**) BSA 20, (**d**) - (**f**) BSA 50, (**g**) - (**i**) BSA 75, (**j**) - (**l**) BSA 100, (**m**) - (**o**) BSA 125 and (**p**) - (**r**) BSA 150.

**Fig. 5:**
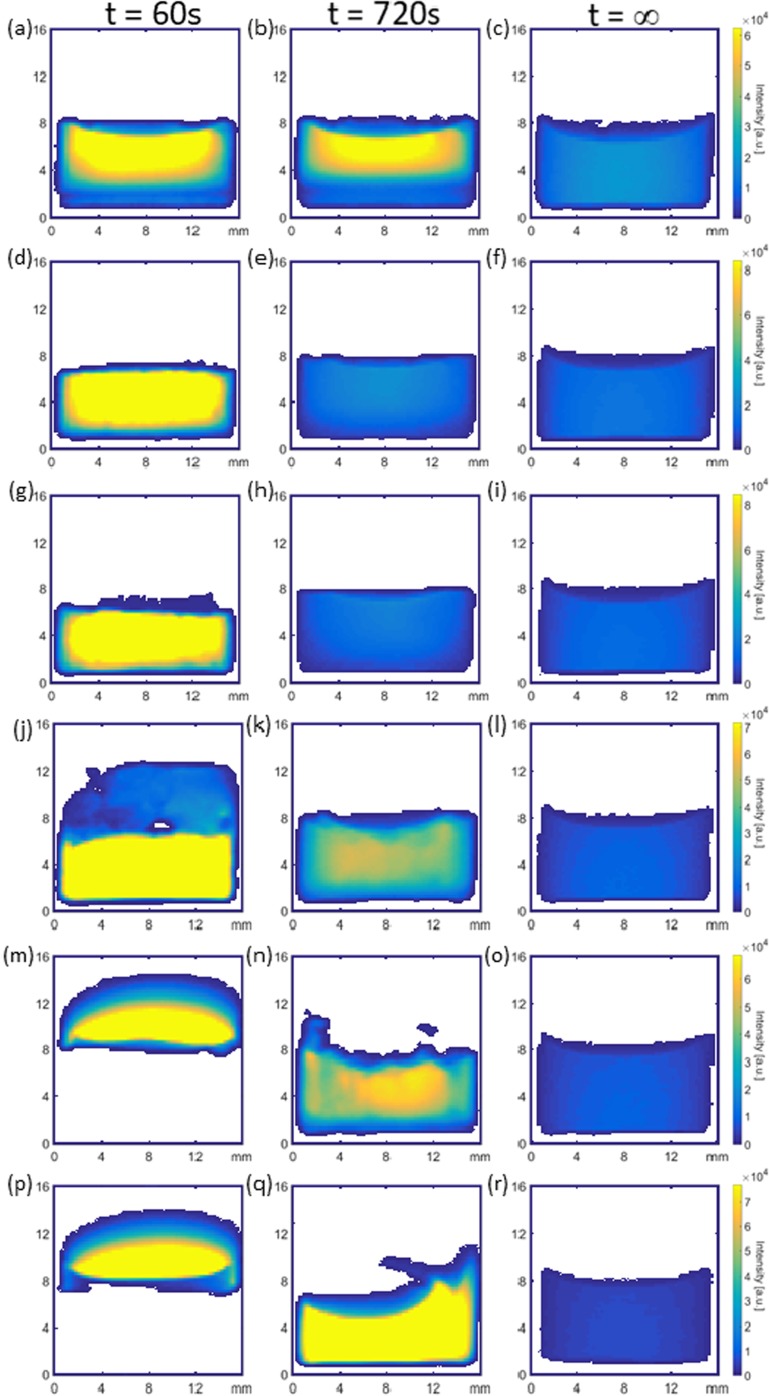
^1^H MRI images at 60 s, 720 s and post swirling (t = ∞) respectively for: (**a**) - (**c**) mAb 20, (**d**) - (**f**) mAb 50, (**g**) - (**i**) mAb 75, (**j**) - (**l**) mAb 100, (**m**) - (**o**) mAb 125 and (**p**) - (**r**) mAb 150.

Figure 4(a-c) shows ^1^H magnetic resonance images for BSA 20 at three different times during the reconstitution process, (the complete set of images are available as supplementary data S4). At t = 60 s post addition of 1 ml of water, two distinct regions of different ^1^H signal intensity are evident: (i) a large upper region of high ^1^H signal intensity (yellow) covering most of the visible image and (ii) a much smaller region at the bottom of the vial with lower overall ^1^H signal intensity (blue green). Region (i) is indicative of water with a small amount of dissolved solids present whereas region (ii) contains a very high amount of dissolved solids. The reason for the low ^1^H signal intensity towards the bottom of the sample vial in the image is due to the T_2_ relaxation contrast introduced into the image by the RARE MR imaging sequence (55). It is worth noting again that the images are a projection of the entire y-axis onto a 2D plane, thus given the cylindrical vial geometry the intensity is expected to be greater in the middle of the vial where a greater volume of ^1^H spins are present. In general, the lower the actual T_2_ value of a ^1^H spin system the less signal will be present in the images. The reason for the region of low ^1^H intensity may be explained by inspection of the visual reconstitution images (see supplementary data S5) which show a rapid breakdown of the lyophilized product and subsequent fall in vertical height as the first drops of liquid water hit the top surface of the product. Rapid dissolution causes a liquid region with higher dissolved solids at the bottom of the vial. Subsequent drops of water then collect on top of this region and this water has much less dissolved solids resulting in a high ^1^H signal intensity given by the yellow color in Fig. 4(a,b). At t = 720 s the MR images show that the lower ^1^H signal intensity region has propagated upwards at the expense of the higher ^1^H intensity region above it, with approximately 1/3 of the sample area now having a lower ^1^H signal intensity; this is a result of Fickian diffusive mixing driven by solute concentration differences and is verified in a later section. A comparison of the MRI data at t = 720 s with visible observations at an equivalent time shows that there are no visible solids present in the bulk liquid at t = 720 s. Following mechanical swirling and replacement of this sample back into the MR scanner a homogeneous distribution of ^1^H signal intensity is restored across the image at t = ∞. Figure 5(a-c) shows a qualitatively similar behavior for mAb 20 with distinct stratification being observed at t = 60 and 720 s along with a homogenous ^1^H signal intensity distribution post swirling at t = ∞.

##### BSA 50–100 mg/ml Samples

Figure 4(d-l) shows the ^1^H images obtained during the reconstitution process for BSA 50, 75 and 100 samples. Again, at t = 60 s, all three sample concentrations display two vertical regions of higher and lower ^1^H signal intensity. The increase in ^1^H signal intensity for BSA 100 relative to BSA 75 is indicative of different spatial concentrations of dissolved solids, with BSA 100 maintaining less dissolved solid upon initial hydration. At t = 720 s two stratified regions are visible but are considerably less discernible than for BSA 20. A homogenous distribution in ^1^H signal intensity is observed post swirling at t = ∞.

##### mAb 50–100 mg/ml Samples

Figure 5(d-l) shows the ^1^H images obtained during the reconstitution process for mAb 50, 75 and 100 samples. At t = 60 s, both mAb 50 and 75 show a homogenously distributed ^1^H signal, indicative of evenly dispersed undissolved solids in a liquid layer. A subtle feature of Fig. 5(d, g) is that the height of the meniscus is significantly reduced when compared to the sample(s) images at t = ∞ (Fig. 5(f, i)). The reason for this is due to the early formation of a liquid/air foam layer that resides on top of the main bulk liquid (this is also supported by the visual observations given in the supplementary information S5). This foam layer is invisible to the RARE MRI sequence due to foam having a short T_2_ value; however as the liquid phase of the foam drains into the main bulk liquid below, the height of the meniscus gradually increases. For mAb 100 at t = 60 s, the bulk signal in the image is similar to mAb 50 and 75 but faint ^1^H intensity shown by dark blue colors above the bulk liquid is indicative of a partially wetted large solid mass. At t = 720 s, there is some evidence of ^1^H intensity inhomogeneity near the corners of the vial in mAb 50 and 75. These samples are in general much more homogenous than their BSA 50 and 75 counterparts. At t = 720 s, mAb 100 shows higher ^1^H intensities when compared to mAb 50 and 75 due to regions of undissolved solid material residing in the bulk liquid. All samples conform to a homogeneous solution after swirling at t = ∞.

##### BSA 125–150 and mAb 100–150 mg/ml Samples

Figure 4(m-r) shows the 2D ^1^H MR imaging results for BSA 125 and 150 while Fig. 5(m-r) shows those for mAb 125 and 150. At t = 60 s, all samples show a high ^1^H signal intensity region at the top of the vial consistent with free water, as well as a region of little to zero signal intensity (white areas) representing undissolved, undisturbed solid product. Small amounts of water penetration down the sides are evident, particularly in the case of BSA 125.

At t = 720 s, a near homogeneous distribution in ^1^H signal intensity is observed for BSA 125 while BSA 150 shows evidence of partial reconstitution (i.e. undissolved solids mixed in a partially reconstituted solution). At t = 720 s, mAb 125 begins to homogenize in physical height but the more intense, less homogeneous ^1^H signal is indicative of large undissolved solid regions within the main liquid bolus. For mAb 150 at t = 720 s, an intense ^1^H signal (yellow color) with a curved meniscus above is also seen; this is indicative of pure water that has penetrated around the sides of the vial and, as it does so, it pushes the solid product vertically upwards so that the undissolved solid mass effectively floats on the liquid below; again these observations are supported by visual observations (supplementary information S5). All samples conform to a homogeneous solution after swirling at t = ∞.

#### Determining the Morphological Steady State of Reconstitution

For all BSA and mAb samples it is important to determine when a reconstituting system has physically reached a morphological steady state following the addition of 1 ml of reconstituting liquid. We define the morphological steady state as one in which there are no distortions to the physical shape of the 2D images. In an attempt to determine this steady state condition objectively we analyze, morphologically, the temporal changes in the physical shape of the ^1^H MR images for each individual sample shown in Figs. 4 and 5 according to the procedure outlined below:

1. All ^1^H images are first thresholded with a single value to produce a binary image containing only ones and zeros.
2. The binary images are then projected along the x-axis to give a single value for each individual image *versus* time.
3. The average value of the last ten images is then taken as a steady state reference value.
4. All numerical values of the previous images are then compared to the steady state value and when the difference is within ±3 units then the system is deemed to have reached steady state.

**Fig. 6:**
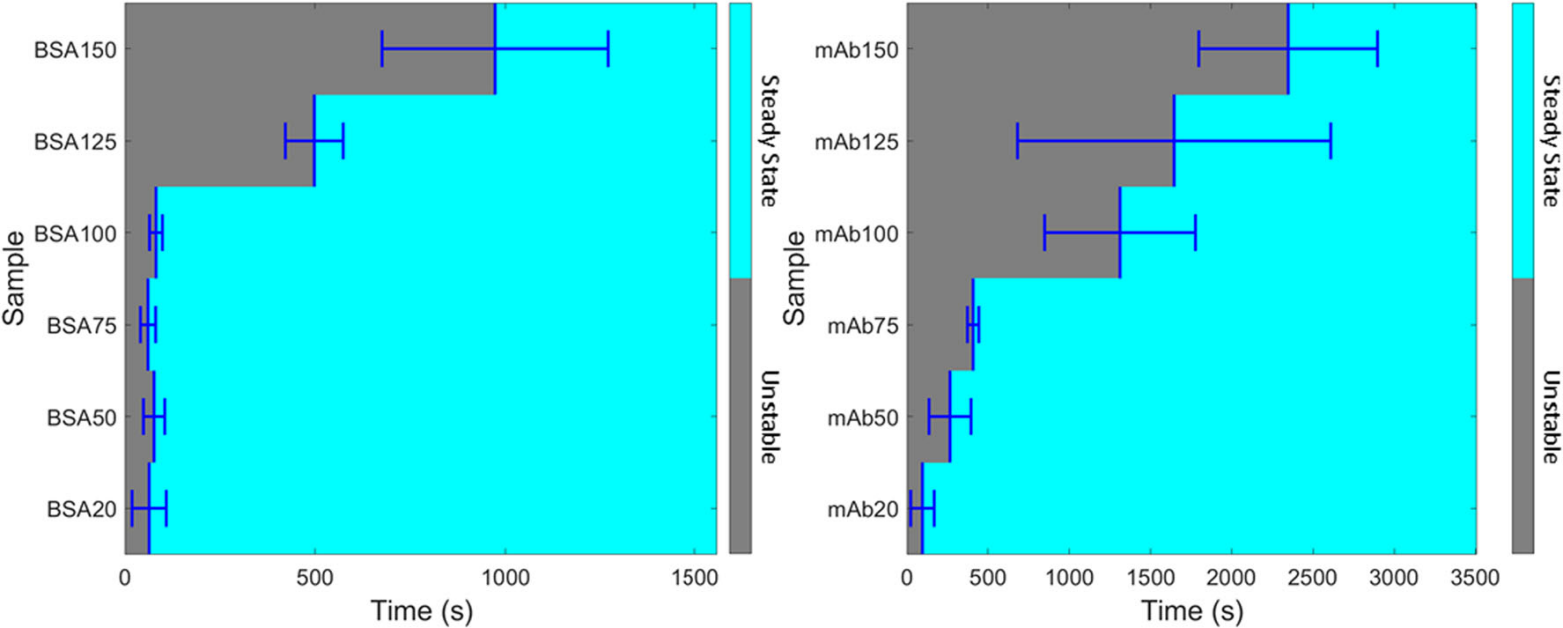
Results from the morphological analysis procedure outlined in (1–6) above for (**a**) BSA and (**b**) mAb images. Grey colors represent times where the morphology of the image has not reached steady state (unstable) and the light blue regions indicate steady state with the average onset of steady state time defined by the dark blue lines. Errors are given as a standard deviation for *n* = 3 samples.

Figure 6 shows the result of this analysis for all BSA and mAb samples and the numerical values extracted from Fig. 6 are shown in Table II. Fig. 6 and Table II generally show that the steady state time increases with increasing protein concentration in line with the visual observations (see supplementary information S5).

**Table II:** Time Points for the Morphology of BSA and mAb Sample Images to Reach Steady State (*n* = 3)

Target Sample Concentration (mg/ml)	BSA steady state time ± SD (s)	mAb steady state time ± SD (s)
20	64 ± 44	96 ± 72
50	77 ± 28	266 ± 128
75	61 ± 20	408 ± 30
100	82 ± 16	1312 ± 464
125	498 ± 76	1645 ± 964
150	973 ± 297	2347 ± 549

The physical interpretation of the results following the morphological analysis of the images is subtle. As no more water enters the system after injection at the start of the experiment, any increase or decrease outside the error boundary (3 units) relative to the reference value is attributed to the physical movement of large clumps of solids and/or foam within the system. These physically displace the liquid to different spatial positions within the field of view of the image. Larger errors in Table II are generally seen for higher concentration samples indicating the movement of large lumps of solids or foam drainage. In general, the reproducibility for BSA samples is good for lower concentration samples but for BSA 125 and 150 a larger error is observed, due to bulk solids/foam movement. The subsequent dissolution behavior for BSA 125 and 150 samples will likely depend upon the sample-to-sample variation in micro cracks present in the initial dry solid product as shown in Fig. 3(c). Further interpretation and consequences of the data presented in Table II and Fig. 6 are presented in “Partitioning of the T_2_ Relaxation Distributions” section where the T_2_ partitioning analysis of the T_2_ relaxation time distributions are discussed.

#### Self-Diffusion Maps from MR Images

**Fig. 7:**
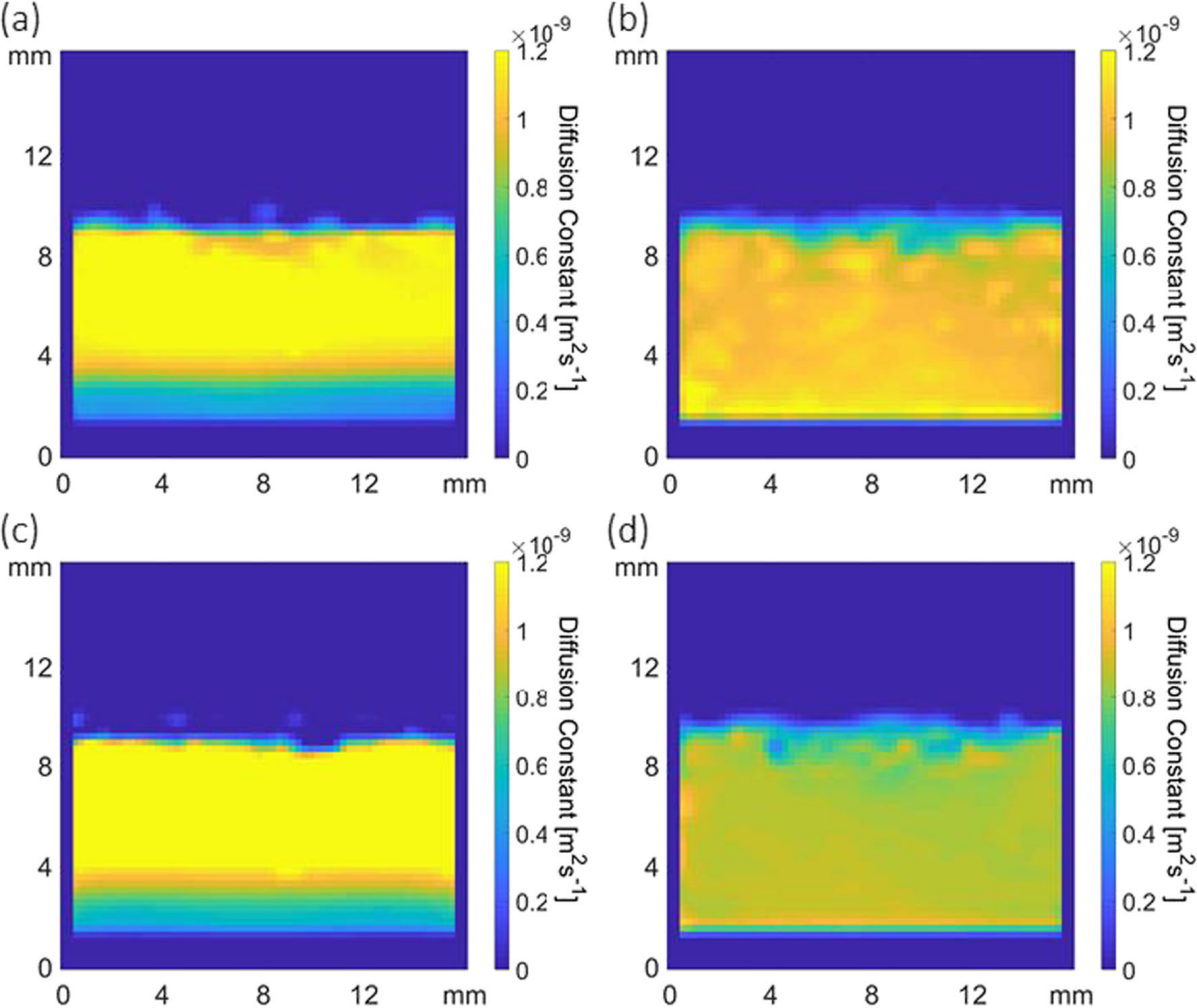
Molecular self-diffusion coefficient ^1^H images of BSA 20 at (**a**) t = 720 s and (**b**) t = ∞, and mAb 20 at (**c**) t = 720 s and (**d**) t = ∞.

In order to investigate the mass transport characteristics of stratified systems discussed in the previous section, diffusion weighted MRI RARE imaging was performed (55). Fig. 7(a) shows a water self-diffusion coefficient map of a BSA 20 sample at t = 720 s and Fig. 7(b) shows a self-diffusion coefficient map for the same sample after swirling. Fig. 7(a) shows two distinct regions, a yellow upper and a blue/green lower, with average self-diffusion coefficients corresponding to 1.2 ± 0.1 × 10^−9^ m^2^ s^−1^ and 0.5 ± 0.1 × 10^−9^ m^2^ s^−1^ respectively. Hence the stratification phenomena observed are due to differences in fluid density causing a difference in water mobility, with the lower layer in the images for BSA 20 being considerably more viscous than the upper layer. The reduced mobility of the water results in a lower T_2_ relaxation time value as the intensity of the ^1^H signal acquired using the RARE imaging sequence is proportional to T_2_ relaxation, with lower relaxation times giving less signal. Fig. 7(b) shows the water self-diffusion map after physical agitation (swirling) of the same sample and clearly shows a more uniform behaviour corresponding to a homogeneously mixed solution of dissolved protein, with an average diffusion coefficient of 1.0 ± 0.1 × 10^−9^ m^2^ s^−1^. A similar interpretation can also be applied to mAb 20 shown in Fig. 7(c) and (d). The average diffusion coefficients of the top and bottom layers in Fig. 7(c) are 1.4 ± 0.1 × 10^−9^ m^2^ s^−1^ and 0.6 ± 0.1 × 10^−9^ m^2^ s^−1^ respectively, while the swirled value average for Fig. 7(d) is 0.87 ± 0.03 × 10^−9^ m^2^ s^−1^. For reference, the diffusion constants for BSA and mAb buffer alone were measured at 1.0 ± 0.1 × 10^−9^ m^2^ s^−1^ and 0.86 ± 0.05 × 10^−9^ m^2^ s^−1^. It is important to point out that when BSA and mAb 20 samples were left in the magnet and continuously imaged over 14 h without removal (and hence no mechanical agitation/swirling) they do produce a homogeneously mixed solution due to diffusive mixing over a long timescale (see supplementary data S6).

### T_2_ Relaxation Time Distribution Data

**Fig. 8:**
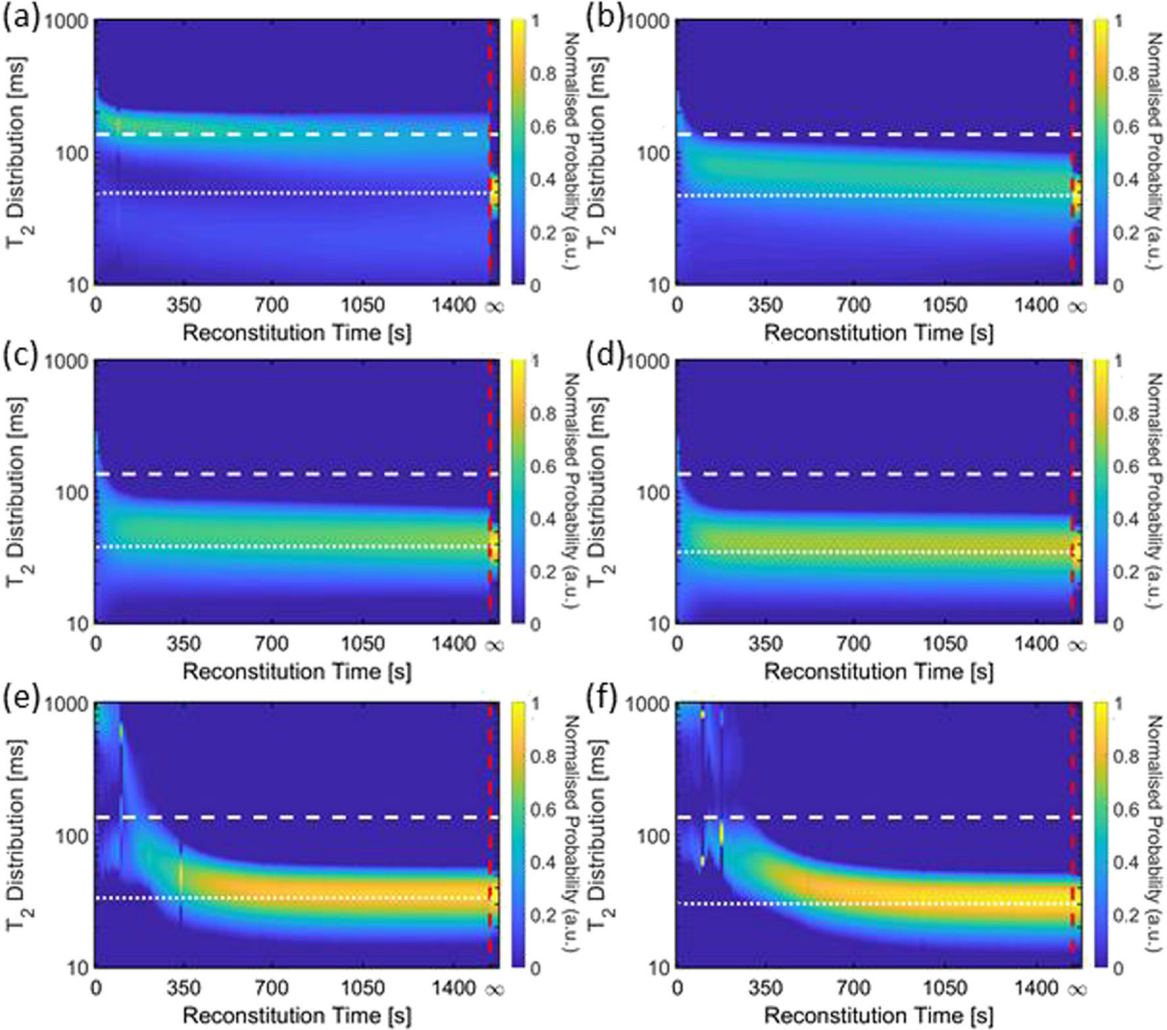
T_2_ probability distributions (normalised to the swirled data at t_inf_) from numerical inversion analysis of one-shot CPMG for t = 0 s to t = 1541 s, and post removal from the magnet followed by swirling and replacement back into the magnet to determine t = ∞ (demarcated by the red dashed line): (**a**) BSA 20, (**b**) BSA 50, (**c**) BSA 75, (**d**) BSA 100, (**e**) BSA 125, and (**f**) BSA 150. The white dashed line indicates the value of t_ref_ and the white dotted lines indicate the value for t_inf._

In order to complement and enhance the observations from the ^1^H 2D imaging data one-shot T_2_ CPMG relaxation time data were acquired for all samples discussed so far. The numerical inversion analysis of the one shot T_2_ relaxation time decays results in a time resolved probability density plot of relaxation time distributions. Fig. 8 shows the complete evolution of normalised T_2_ relaxation time distributions for all BSA samples after addition of 1 ml of water. The horizontal axis in these plots gives the reconstitution time, the vertical axis denotes the actual T_2_ relaxation time parameter obtained from the numerical inversion analysis; the color scale bar to the right of each plot represents the probability of each particular relaxation time. Table III shows the weighted t_av_ values extracted from both BSA and mAb sample T_2_ distributions at three different time points. In general, BSA samples have final T_2_ values which vary from 50 to 30 ms, while mAb samples have a tighter range of 49 to 36 ms, as concentration increases from 20 to 150 mg/ml. Considering some general features of the T_2_ shown in Fig. 8, for BSA 20 a distinct two component behaviour in the T_2_-distribution is seen (Fig. 8(a)) and is attributed to the stratified layers revealed by the ^1^H images shown earlier in Fig. 4.

**Fig. 9:**
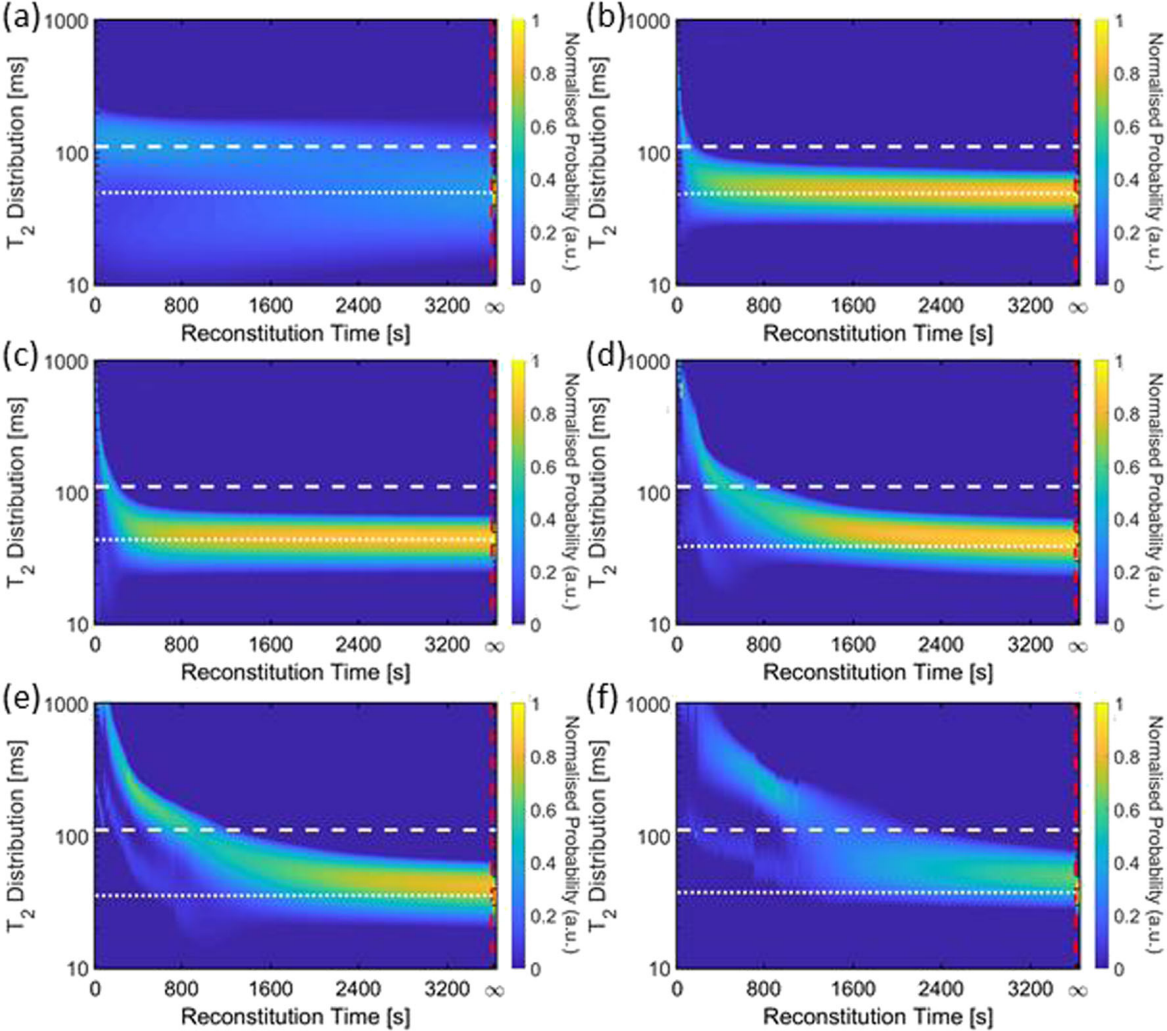
T_2_ probability distributions (normalised to the swirled data at t_inf_) from numerical inversion analysis of one shot CPMG for t = 0 s to t = 3620 s, and post removal from the magnet followed by swirling and replacement back into the magnet to determine t = ∞ (demarcated by the red dashed line): (**a**) mAb 20, (**b**) mAb 50, (**c**) mAb 75, (**d**) mAb 100, (**e**) mAb 125, and (**f**) mAb 150. The white dashed line indicates the value of t_ref_ and the white dotted lines indicated the value for t_inf._

**Table III:** Weighted Average T_2_ Values from BSA and mAb Samples in Figs. 8 and 9 Respectively (*n* = 3)

Sample	t = 60 s	t = 720 s	t = ∞
	t_av_ ± SD (ms)	t_av_ ± SD (ms)	t_inf_ ± SD (ms)
BSA buffer	120 ± 10	100 ± 10	51 ± 1
BSA 20	120 ± 10	85 ± 9	50 ± 1
BSA 50	71 ± 1	55 ± 1	47 ± 1
BSA 75	50 ± 2	44 ± 2	38 ± 1
BSA 100	44 ± 2	37 ± 1	34 ± 1
BSA 125	470 ± 50	36 ± 1	34 ± 1
BSA 150	760 ± 70	37 ± 4	30 ± 1
mAb buffer	110 ± 10	100 ± 10	60 ± 1
mAb 20	90 ± 10	74 ± 7	49 ± 1
mAb 50	91 ± 4	52 ± 2	48 ± 1
mAb 75	140 ± 20	48 ± 1	45 ± 1
mAb 100	600 ± 200	68 ± 3	39 ± 1
mAb 125	1200 ± 100	65 ± 3	36 ± 1
mAb 150	1300 ± 500*	248 ± 8*	36 ± 2

* for mAb 150 at t = 60 and 720 s, *n* = 2 due to limited samples

For BSA 50–100 samples (Fig. 8(b-d)) the T_2_ distributions quickly approach the t_inf_ value of the swirled solution denoted on the figure by the white dotted line. For BSA 125 and 150 samples (Fig. 8(e,f)), a distinct chaotic/turbulent behaviour of the T_2_ values for 0 < t < 350 s is evident and is attributed to bulk movement of liquid and solids, which is corroborated by both the ^1^H MR images and visual observations for equivalent samples.

Figure 9 shows the equivalent T_2_ relaxation time distributions for mAb samples for t = 0 to t = ∞. Similar features to the BSA samples are observed: for mAb 20 at early times a two component behaviour is seen indicating distinct stratification (see Fig. 5(a,b)). T_2_ relaxation time distributions for mAb 50, 75 and 100 samples rapidly approach the swirled value of t_inf_. Chaotic/turbulent regimes are also seen in mAb 125 and 150 samples for t ~ 30–250 s. Note that for mAb samples the rate of change of the T_2_ relaxation time with reconstitution time decreases with increasing protein concentration.

#### Partitioning of the T_2_ Relaxation Distributions

**Fig. 10:**
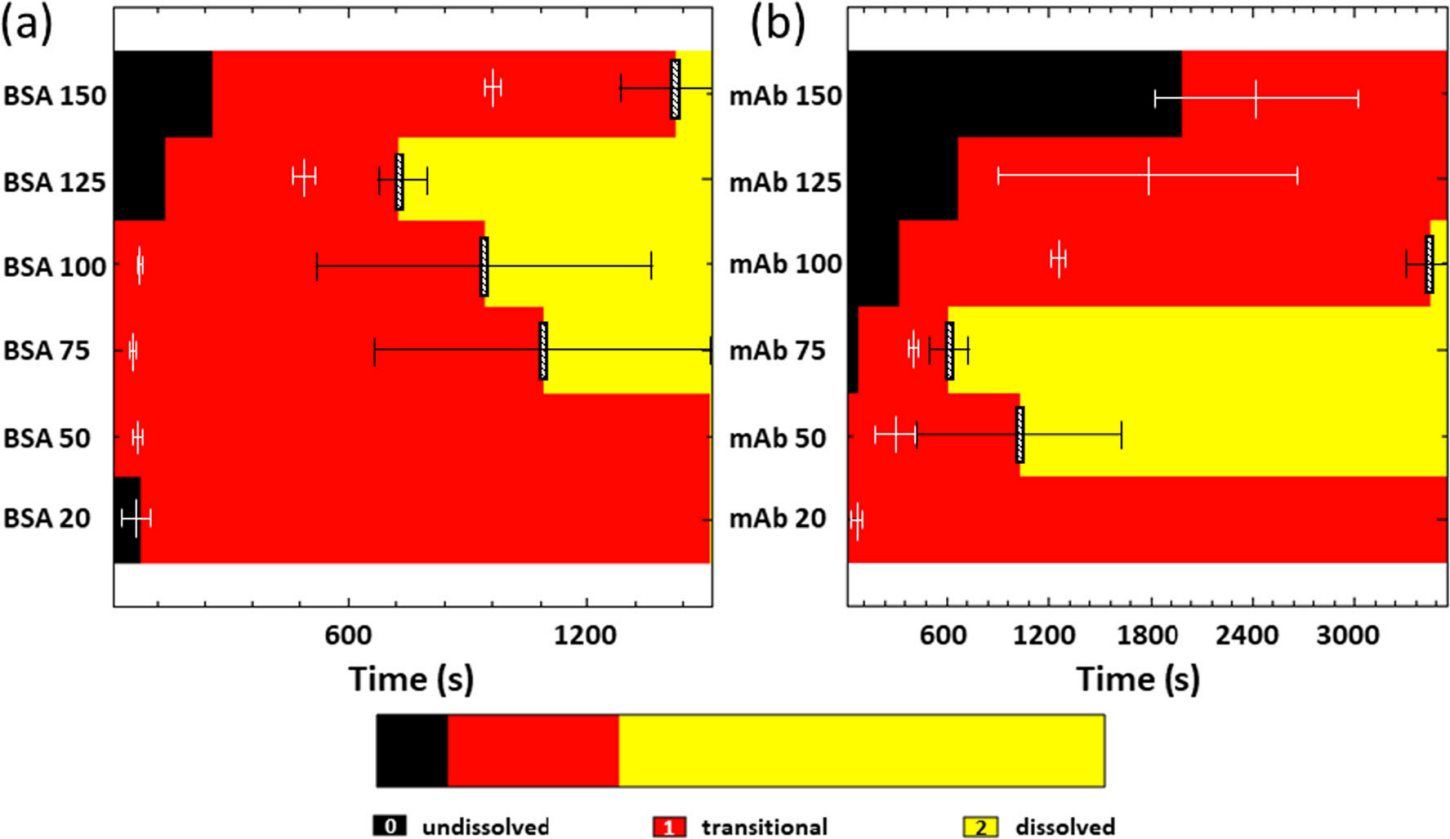
Schematic 1D maps based upon the partitioning protocol for (**a**) BSA samples and (**b**) mAb samples. Three values for the scale bar are used representing undissolved (black), transitional (red), and dissolved (yellow) time points during the reconstitution process (see “T_2_ Partitioning” section). The white vertical lines represent the point at which the morphology of the liquid image has reached steady state and the black hatched bar indicates the time point at which the reconstitution is deemed complete.

In an attempt to automate/objectify the analysis procedure to determine reconstitution time from T_2_ data, the T_2_ relaxation time distributions shown in Figs. 8 and 9 were analyzed according to the partitioning conditions outlined in “T_2_ Partitioning” section. Fig. 10 shows the results of this analysis.

In addition, the data from Fig. 6 are also included in Fig. 10 (as white vertical bars) and show the time when the morphology of the liquid in the ^1^H MR images has reached steady state. Some general features for the interpretation of Fig. 10 are as follows:

1. Black colored regions signify an average T_2_ value that is greater than the buffer solution reference (condition (iv) in “T_2_ Partitioning” section: t_av_ > t_ref_) and are indicative of solutions with a limited amount of dissolved solid material and/or large clumps of undissolved solids. Hence, black regions are referred to as *undissolved* systems. The error in the boundaries from black to red are not included on Fig. 10 but may be found in Table S1 of supplementary information.
2. The position of the white vertical bar (and their associated error boundaries) indicates the time point when the morphology of the reconstituting system has reached steady state (data from Table II).
3. Red colored regions in Fig. 10 correspond to a system where the weighted average T_2_ values are transitioning (condition (v) in “T_2_ Partitioning” section: t_inf_ + 3σ < t_av_ < t_ref_) and represents a system where solids are continuously dissolving into the bulk liquid or are indicative of fully dissolved liquid-liquid stratified systems. This region is referred to as *transitional*.
4. Yellow colored regions in Fig. 10 correspond to condition (vi) in “T_2_ Partitioning” section, i.e. t_av_ < t_inf_ + 3σ. The black hatched vertical bar indicates the time point where this occurs and is defined as the point where the reconstitution can be considered homogeneous and complete within the sample to sample error (*n* = 3). Yellow regions are defined as *dissolved*.

**Fig. 11:**
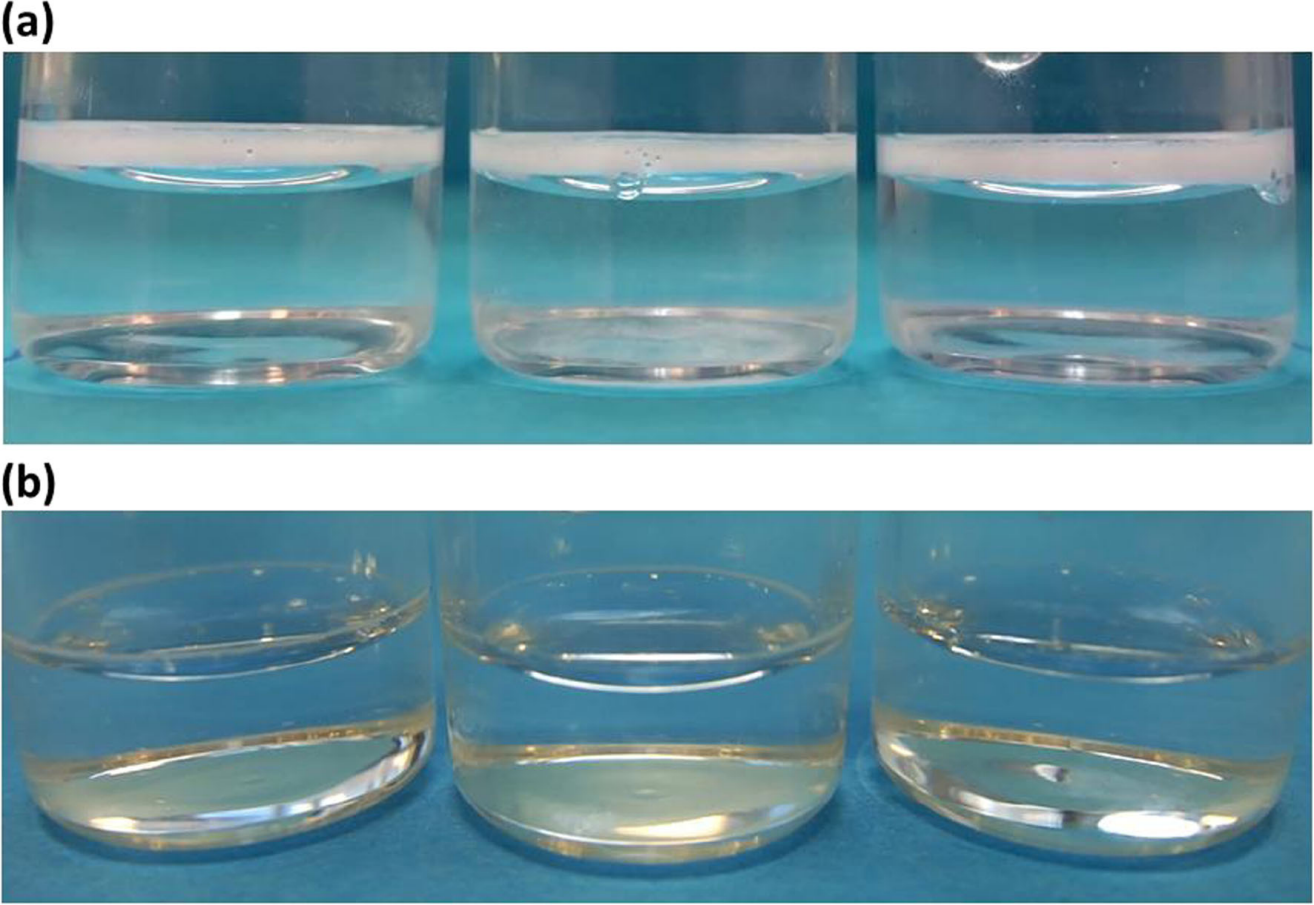
Visual image of three buffer vials from the same batch at t = 90 s after injection for (**a**) BSA and (**b**) mAb.

Two further points are worth noting in relation to (1) and (2) above. The total time of the reconstitution experiment for both BSA and mAb samples shown in Fig. 10 is 26 min and 60 min respectively. Visual observations (see also Table I) of reconstituting reference buffer solutions of both BSA and mAb, show that these samples are fully reconstituted before t < 90 s (allowing for error) as shown in Fig. 11. This time point can thus be used as a control/reference to compare with the protein containing samples. Hence, the T_2_-relaxation data of the buffer samples can be used to refine the reconstitution time.

**Fig. 12:**
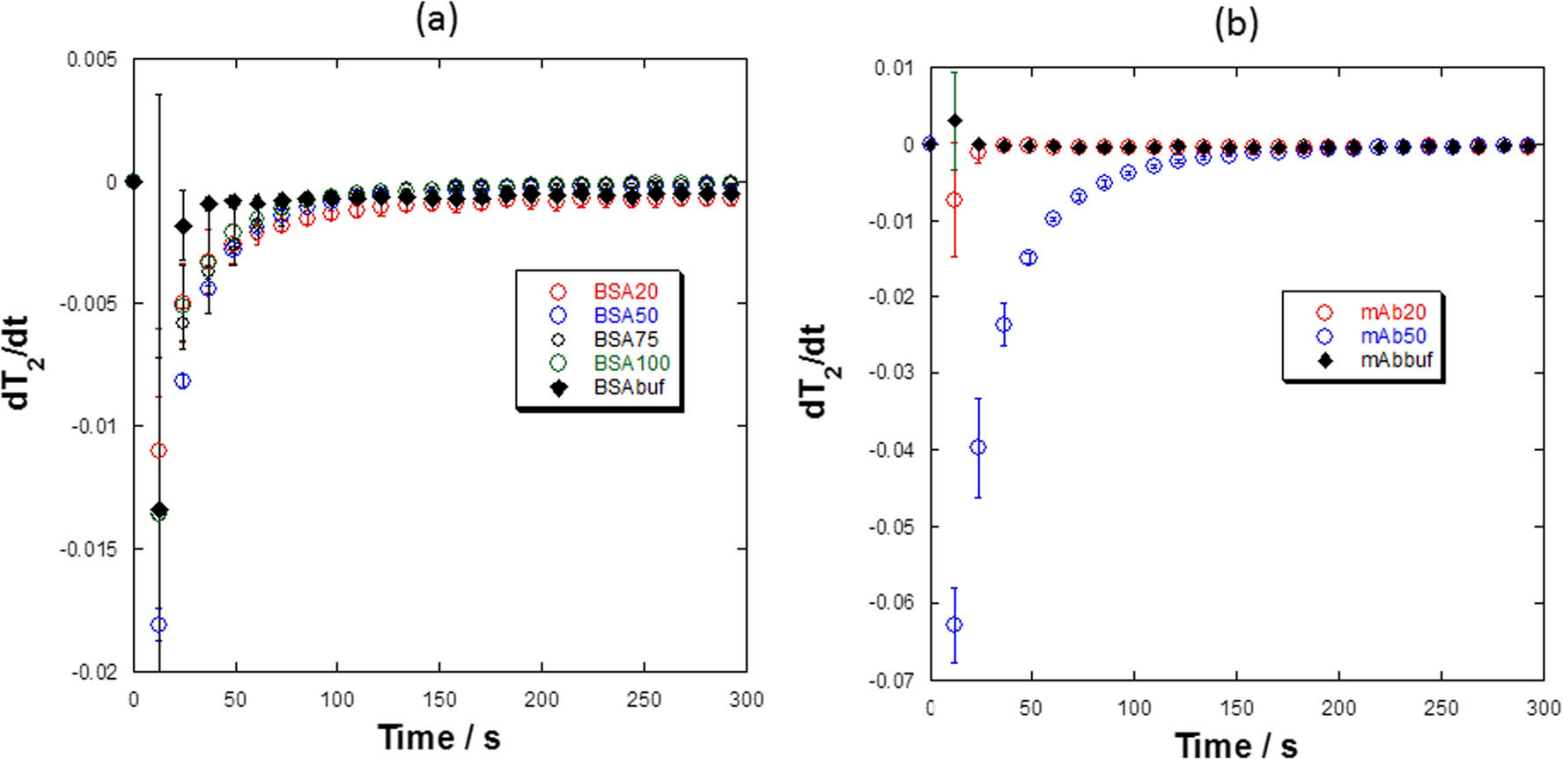
Plots of $\left(\frac{d{t}_{av}}{dt}\right)$ *vs*. time for (**a**) BSA 20–100 and BSA buffer samples; (**b**) mAb 20 and mAb 50 samples along with mAb buffer. Three (*n* = 3) independent samples were analyzed and the error bars represent a standard deviation. We note that buffer solutions also show distinct bi-modal T_2_ behaviour due to liquid-liquid stratification.

Returning to Fig. 10, BSA and mAb samples that have reached a morphological steady state (white vertical bars, Fig. 10) by t ~ 240 s and are followed by (or within) red colored regions are generally deemed *transitional*, e.g. BSA 20, 50, 75 and 100, and mAb 20 and 50 samples. The figure of t = 240 s is determined from the visual reconstitution data for protein samples which for BSA 20, 50, 75 and 100, and mAb 20 and 50 samples (see supplementary data S5, S7) shows that there are no visible solids in the bulk solutions by t = 240 s; these would then be characterized as being fully reconstituted (fully dissolved) from a visual determination. The fact that these solutions are deemed (from T_2_ partitioning), as *transitioning*, is, in most part, a result of liquid-liquid stratification from two fluid layers with different densities with distinct (bi-modal) T_2_ values, which causes the weighted average T_2_ to be higher than that expected for a homogenous system. As time proceeds the stratified layers slowly mix and eventually give a homogenous solution with a mono-modal, symmetric, T_2_ relaxation time distribution (see supplementary data S6). Samples that are deemed *transitioning* from T_2_ partitioning alone and have also reached a morphological steady state by t ≤ 240 s are subject to an additional analysis, called differential T_2_ analysis, that allows a more definitive reconstitution time to be determined. Once this time is calculated, these samples can be considered as *fully dissolved*. The differential T_2_ analysis is described as follows:

1. The t_av_ data extracted from Figs. 8 and 9 may be differentiated, $\left(\frac{d{t}_{av}}{dt}\right)$, with respect to time to obtain plots showing how $\left(\frac{d{t}_{av}}{dt}\right)$ varies with reconstitution time (see Fig. 12).

(2) The $\left(\frac{d{t}_{av}}{dt}\right)$ values for the buffer solution are also calculated and plotted
(3) An average value $\left(\frac{d{t}_{av}}{dt}\right)$ and standard deviation (σ) for the reference buffer solution(s) is then calculated between two time limits: (i) a lower t = 90 s limit determined from visual observations of reference buffer samples and (ii) an upper limit of t = 240 s corresponding to the time the visual reconstitutions for protein containing samples are deemed complete (see supplementary Figure S7).
(4) The value(s) calculated in (3) are then compared to the $\left(\frac{d{t}_{av}}{dt}\right)$ data from the protein samples in (1) up to t = 300 s and when those values are greater than or equal to $\left(\frac{d{t}_{av}}{dt}-3\sigma \right)$ for the reference buffer solution, then the corresponding time point along the horizontal axis is noted and assigned the time for reconstitution.

The results of this T_2_ differential analysis are shown in Fig. 12 and the reconstitution times are given in Table IV. A reasonable agreement for BSA samples (given the errors associated with both visual and MRI determined reconstitution times) is evident with a closer agreement for mAb samples. For BSA samples it is evident that $\left(\frac{d{t}_{av}}{dt}\right)$ for BSA 20 is the slowest to approach $\left(\frac{d{t}_{av}}{dt}-3\sigma \right)$ of the reference buffer samples, taking around 159 s and BSA 100 is the quickest to approach the buffer reference yielding a reconstitution time 69 s. The reconstitution time resulting from the T_2_ differential analysis for BSA 20 is somewhat longer than the visual reconstitution time but is within the error associated with the differential T_2_ analysis. It is likely that the observed stratification and hence skewness of the bi-modal T_2_ distribution still has some impact in this case. For BSA concentrations of 50–100 mg/ml the reconstitution times determined using the T_2_ data are generally shorter than those recorded visually, especially for BSA 100. Despite this, it is suggested that the differential T_2_ analysis represents a more objective reconstitution time as it is less influenced by very small amounts of undissolved solid particles suspended in the meniscus foam or particles that are stuck to the edge of the vial that, subjectively, may be deemed important or unimportant by an operator. Examples of these observations can be seen in the visual reconstitution videos (see supplementary information S5) where occasionally a small amount of solid material results in a much longer (subjective) visual reconstitution time, while having limited effect on the average T_2_. This is further supported by the reduced standard deviations in the MRI data compared to the visual observation data.

**Table IV:** A Comparison of Average Reconstitution Times from MRI Data and Visual Observation (*n* = 3)

Sample	MRI ± SD (s)	Visual ± SD (s)
BSA 20	159^a^ ± 113	90 ± 20
BSA 50	93^a^ ± 7	140 ± 50
BSA 75	102^a^ ± 0	220 ± 60
BSA100	69^a^ ± 30	260 ± 40
BSA 125	720 ± 60	1300 ± 100
BSA 150	1520 ± 138	2100 ± 100
mAb 20	32^a^ ± 7	130 ± 30
mAb 50	202^a^ ± 19	260 ± 40
mAb 75	600 ± 158	600 ± 100
mAb 100	3420 ± 172	3000 ± 900
mAb 125	5000^b^ ± 200	6500 ± 400
mAb 150	6480^b,c^	10,000 ± 1000

^a^– denotes a reconstitution time from differential, $\left(\frac{d{t}_{av}}{dt}\right)$, T_2_ analysis. Note: error bars for differential T_2_ analysis were calculated as a standard deviation (*n* = 3)

^b^– acquisition time for mAb 125 and 150 was extended to t > 4 h to allow complete reconstitution

^c^*n* = 1 for mAb 150 due to limited samples

Of particular interest is the physical interpretation of the collective analysis from the different types of magnetic resonance data and subsequent analysis presented here, i.e. steady state morphological analysis, T_2_ partitioning, and differential T_2_ analysis. For example, consider again the BSA 100 sample in Fig. 10(a): the white vertical bar determined from morphological image analysis is located at t = 82 s (see Fig. 6(a) and Table II) and this time is below the upper, fully reconstituted, limit of 240 s pre-defined by the visual observations of the reconstitution process. As the white vertical bar in Fig. 10(a) is below the 240 s limit a differential analysis of the average T_2_ is permitted to refine the estimate of the reconstitution time. An estimate of the value of the reconstitution time from T_2_ partitioning alone is given at the interface of the red and yellow regions if such a region is present in the data, i.e. a system that changes from *transitioning* to a *dissolved* system; for BSA 100 this occurs around t ~ 16 min (960 ± 510 s), which is clearly well above both the differential T_2_ analysis and visual reconstitution times given in Table IV. Hence, the differential analysis, where appropriate, of the T_2_ data yields a more realistic figure (relative to the visual reconstitution times) for the reconstitution time, which is reasonable given the errors associated with both measurements evident in Table IV. The differential analysis of t_av_ described above is not valid when the position of the steady state white vertical line in Fig. 10 is greater than the upper t = 240 s limit determined from the visual observations, e.g. BSA 125–150 and mAb 75–150 samples. In these cases, a definitive reconstitution time is only determinable when the solution has reached a *dissolved* state as indicated by the position of the black hatched bar in Fig. 10. The mAb 125 and 150 samples are not deemed to have reconstituted by T_2_/MRI methods at t = 60 min respectively. Note: if appropriate, the T_2_-differential analysis takes precedence over T_2_-partitioning for the assignment of a final reconstitution time.

### Quantifying Protein Concentrations with MRI

**Fig. 13:**
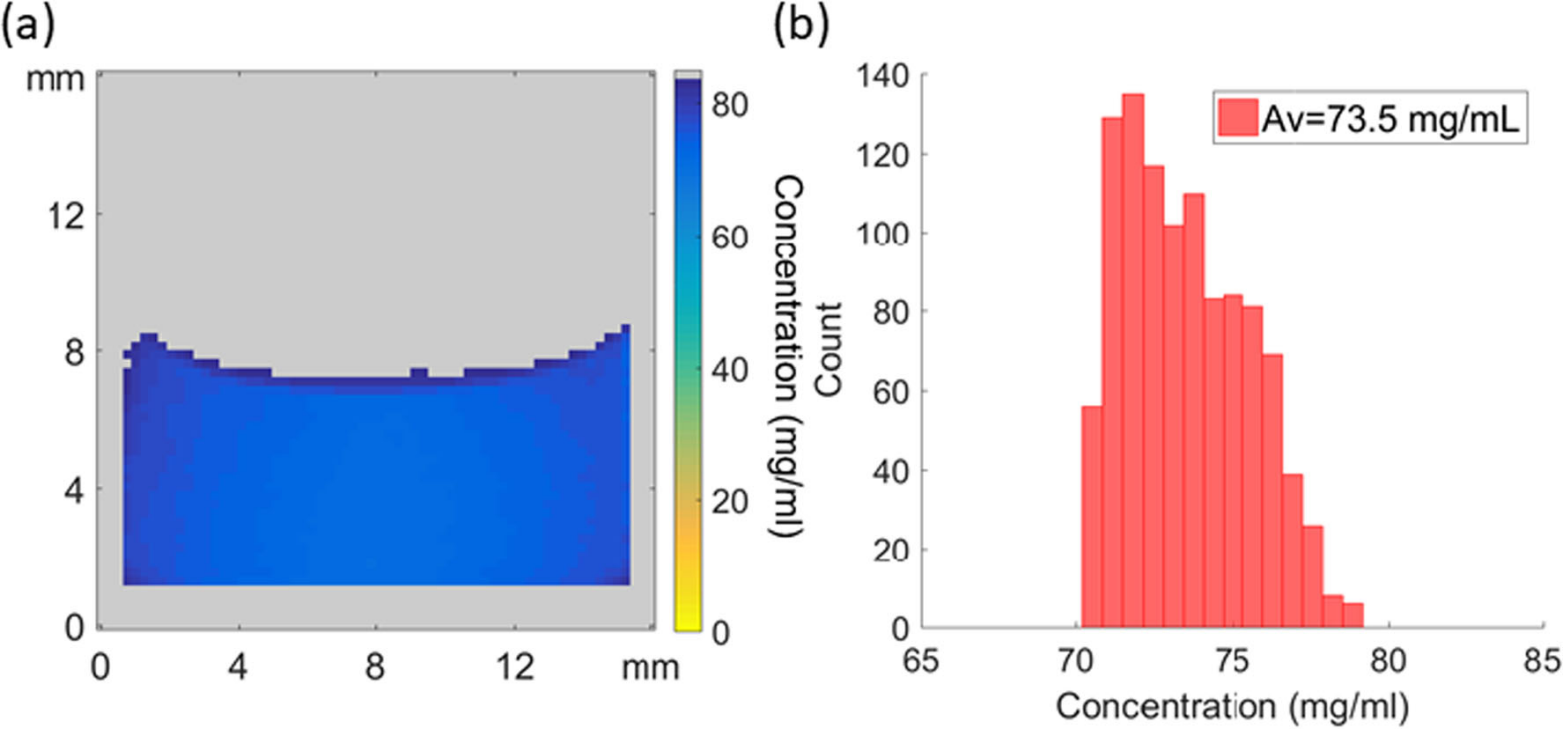
(**a**) Spatially resolved protein concentration maps post swirling, the color bar has been reversed to indicate that low concentrations would be seen as high intensity on the MR images in Figs. 4 and 5 and conversely high concentrations would have low intensities in standard ^1^H imaging. (**b**) Resulting histogram for a boxed area (avoiding the meniscus and edges) for mAb 75.

Magnetic resonance imaging is a quantitative technique in that the amount of signal from a ^1^H spin system is directly proportional to the number of ^1^H nuclei present as long as both T_1_ and T_2_ relaxation contrast in the system are accounted for (56). The ^1^H signal in all experiments presented here is dominated by water as it is in vast excess in terms of concentration and hence the signal from the protein ^1^H resonances give a negligible contribution in the ^1^H RARE images and corresponding T_2_ relaxation data. T_1_ relaxation contrast for the images shown here can be neglected because the recycle time of the experiment was chosen to ensure that >95% of the available ^1^H signal is recovered before subsequent excitation. T_2_ relaxation time contrast can be accounted for if the T_2_ relaxation times of each individual pixel is known, however, this is a time consuming measurement. In order to relate the ^1^H image water pixel intensity at t = ∞ to the dissolved protein concentration, a calibration method (see experimental section and supplementary information S1-S3) was developed to allow the final protein concentration maps to be determined for the images at t = ∞. Figure 13 shows an example protein concentration map at t = ∞ calculated using the procedure outlined in “Protein Concentration Calibration Procedure using MRI” section along with a histogram of concentration values determined by MRI. Table V summarises the results from final swirled concentration measurements for all samples and those independently evaluated from exactly the same samples using the A280 peak in UV absorbance spectroscopy.

**Table V:** Statistical Analysis of a Boxed Area (Avoiding Edges and Meniscus) of the Spatially Resolved Protein Concentration Maps

Formulation	UV Spectroscopy Concentration ± SD (mg/ml)	Average Box Area Concentration ± SD (mg/ml)
BSA 20	20 ± 2	18.8 ± 0.5
BSA 50	51 ± 2	54 ± 1
BSA 75	79 ± 3	82 ± 2
BSA 100	104 ± 4	104 ± 2
BSA 125	124 ± 3	125 ± 4
BSA 150	159 ± 8	167 ± 5
mAb 20	19 ± 2	18.5 ± 0.4
mAb 50	49 ± 2	50.0 ± 0.8
mAb 75	75 ± 1	74 ± 2
mAb 100	105 ± 1	105 ± 2
mAb 125	128 ± 9	119 ± 2
mAb 150	153 ± 4	144 ± 2

**Fig. 14:**
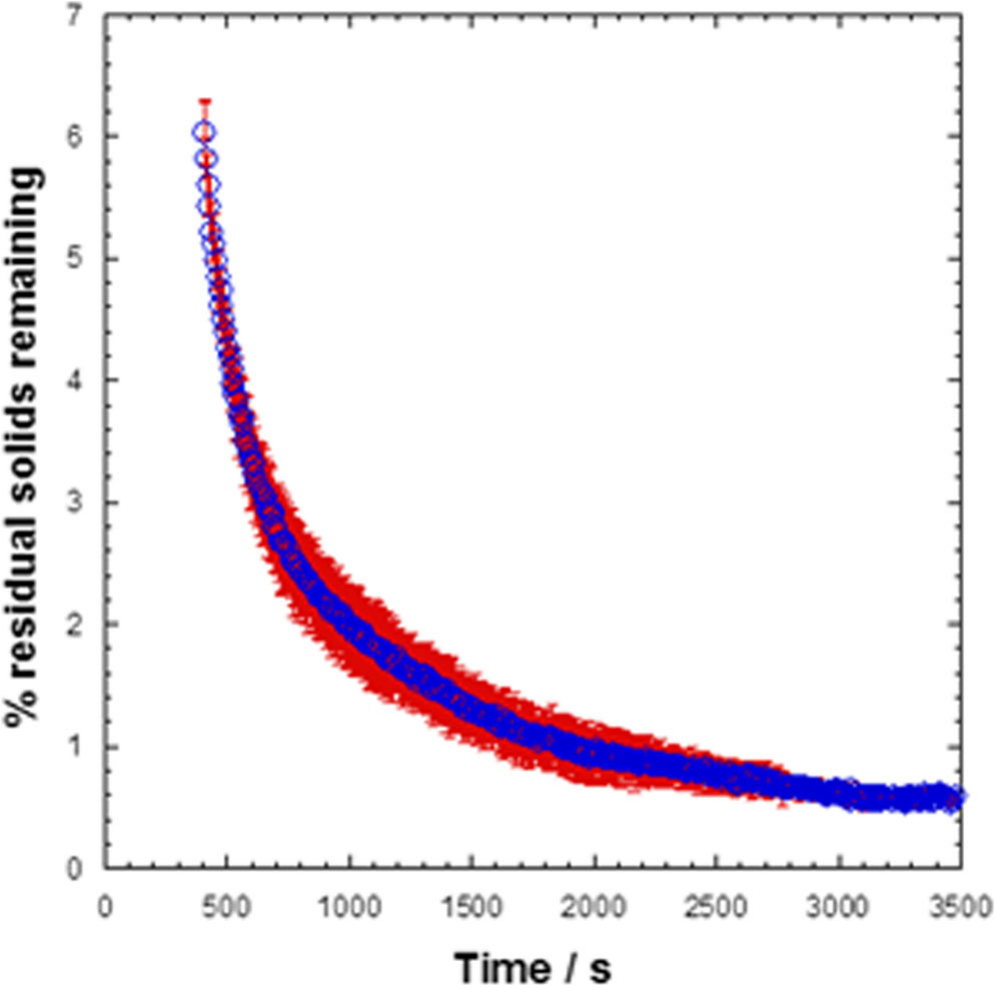
Plot of the variation in percentage remaining solids content *versus* time for mAb 75.

The MRI concentration calibration method can also be used to assess the undissolved solids content for homogeneous samples that have reached steady state. Here we assume that the final swirled sample at t = ∞ represents a state with no remaining solids, which we define as 0% and that any differences in ^1^H signal intensity are due to remaining undissolved solids. Crucially, this method does not work when liquid stratification is present, as seen in many low concentration samples, and is only valid when a reconstituting system has reached a steady state as indicated by the appearance of the white bars in Fig. 10. For example, the mAb 75 sample shows negligible evidence of stratification (see Fig. 5(h)) and has been determined from morphological analysis to have reached a steady state at t = 408 ± 30 s. Using the calibration data described in the experimental section and supplementary information S1-S3 it is possible to estimate the remaining solids concentration for the mAb 75 sample. The results of this analysis are shown in Fig. 14 and indicate that at t = 400 s approximately 6% solids remain, with this value decreasing to 0.5% by t = 3500 s at the end of the experiment, which within experimental error, indicates that the mAb 75 sample can be considered as fully reconstituted. Hence, with suitable calibration of known samples, the ^1^H signal intensity alone of a homogeneous steady state reconstitution system can be used to estimate the remaining solid contents with time.

## Conclusions and Outlook

MR imaging coupled with an objective analysis of T_2_ relaxation time distributions has been applied to two different lyophilized products: a model BSA system and a mAb sample with protein concentrations varying from 20 to 150 mg/ml in each case. A quantitative morphological analysis of 2D ^1^H RARE images coupled with partitioning/differential analysis of the T_2_ relaxation data gives an objective determination of reconstitution times which can be compared with visual analysis. The results for low concentration BSA protein samples <100 mg/ml are in reasonable agreement with visual observation times though large differences are attributed to the subjective nature of the manual/visual reconstitution method with regard to undissolved solids. A good agreement is seen when comparing the reconstitution times from T_2_ data and visual observations for mAb samples. MR diffusion imaging has further revealed that liquid stratification occurs, especially for low protein concentration samples (e.g. 20–50 mg/ml), giving rise to two distinct liquid layers with different viscosities and water self-diffusion coefficients. For BSA and mAb 20 samples, continuous MR imaging for over 14 h showed that these layers eventually form a homogenous mixture with mass transfer being limited by molecular self-diffusion processes. At higher protein concentrations (100–50 mg/ml) for both BSA and mAb samples, stratification is less apparent. The discovery of liquid stratification prevented a simple (partitioning) analysis of the T_2_ data to determine reconstitution times for certain samples. Despite stratification phenomena, giving long periods of *transitional* average T_2_ behaviour, a differential analysis of the T_2_ data was shown to be appropriate in determining a more realistic reconstitution time. An important consideration in this was the use of morphological analysis of the 2D ^1^H images to reveal a unique time point at which these systems can be deemed to have reached a steady state relative to a reference buffer solution. Quantitative protein concentrations of the final swirled solutions can be determined from the ^1^H MR image intensities as long as MR calibration procedures of known phantoms are determined under the same experimental MRI parameters used to image the reconstitution process. The results from MRI determined protein concentrations are in excellent agreement with those from standard UV-vis analysis. At present the MRI protein quantification method only allows us to determine the protein concentration from a homogenous 2D image and is thus not applicable to liquid-liquid stratified systems. This is because the current calibration data is based upon the addition of a minimum of 1 ml of water and subsequent dilution thereafter. In order to relate the ^1^H image intensity of stratified systems to the absolute protein concentration, further calibration experiments are required where additional solids are added to a fixed amount of water, e.g. 1 ml. A demonstration of how the magnetic resonance calibration data can also be used to estimate the remaining solids content of a *dissolved* homogenous system (e.g. mAb 75 was shown. Further refinements of the bi-modal T_2_ behaviour data from stratified systems is ongoing in an attempt to estimate remaining solids content from stratified systems. Finally, the ^1^H single shot CPMG technique for both reconstitution time and final concentration determination could be translated to an inexpensive low field instrument, and with further investigation could become a validated QC test in industry and clinical settings. Future work will focus on how the magnetic resonance imaging and T_2_ relaxation data described here can be combined with both *in situ* porosity measurements of the lyophilized solid product and solid state NMR studies, in an attempt to understand the underlying physicochemical mechanisms governing reconstitution times for lyophilized drug products with different excipient formulations and protein concentrations.

## Electronic supplementary material

ESM 1 (MP4 19,785 kb)

ESM 2 (MP4 60,514 kb)

ESM 3 (MP4 3822 kb)

ESM 4 (MP4 9435 kb)

ESM 5 (MP4 9839 kb)

ESM 6 (MP4 7799 kb)

ESM 7 (MP4 21,071 kb)

ESM 8 (MP4 42,749 kb)

ESM 9 (MP4 3434 kb)

ESM 10 (MP4 3207 kb)

ESM 11 (MP4 11,728 kb)

ESM 12 (MP4 13,810 kb)

ESM 13 (MP4 13,600 kb)

ESM 14 (MP4 14,117 kb)

ESM 15 (MP4 205 kb)

ESM 16 (MP4 4477 kb)

ESM 17 (AVI 17951 kb)

ESM 18 (AVI 15531 kb)

ESM 19 (AVI 15035 kb)

ESM 20 (AVI 16202 kb)

ESM 21 (AVI 15498 kb)

ESM 22 (AVI 18570 kb)

ESM 23 (AVI 18336 kb)

ESM 24 (AVI 39419 kb)

ESM 25 (AVI 30780 kb)

ESM 26 (AVI 31019 kb)

ESM 27 (AVI 36108 kb)

ESM 28 (AVI 37401 kb)

ESM 29 (AVI 38254 kb)

ESM 30 (AVI 18990 kb)

ESM 31 (DOCX 4206 kb)
