# Supplementary material for: Application of Magnetic Resonance to Assess Lyophilized Drug Product Reconstitution
Source: Pharm Res. 2019 Mar 22;36(5):71. doi: 10.1007/s11095-019-2591-x (PMC6430757; doi:10.1007/s11095-019-2591-x)
Supplement: Supplementary file 31 — (DOCX 4206 kb) [file 11095_2019_2591_MOESM31_ESM.docx]

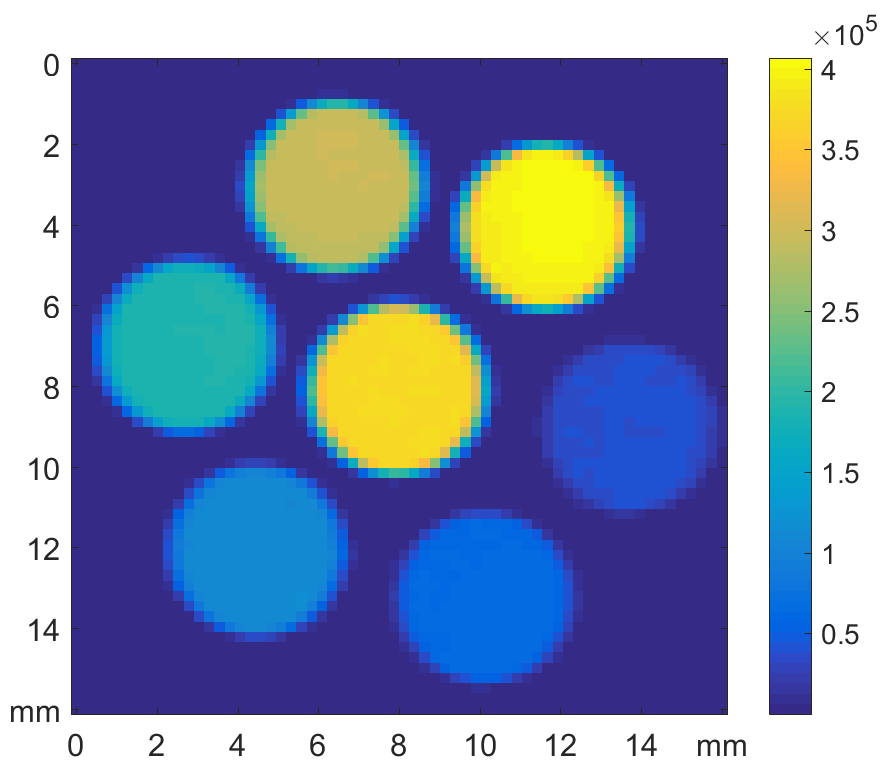

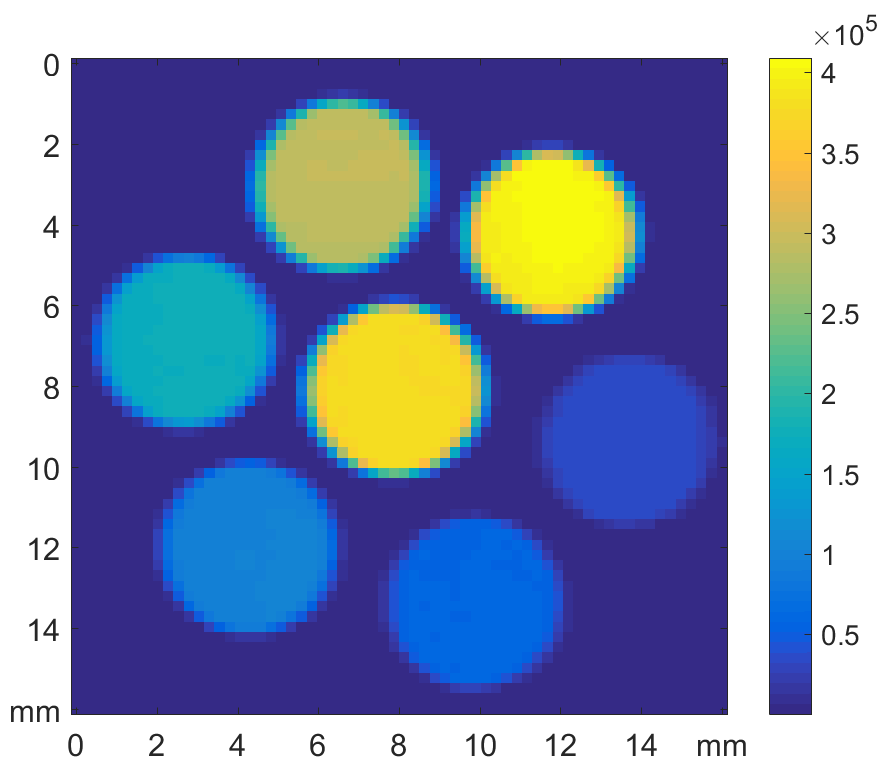

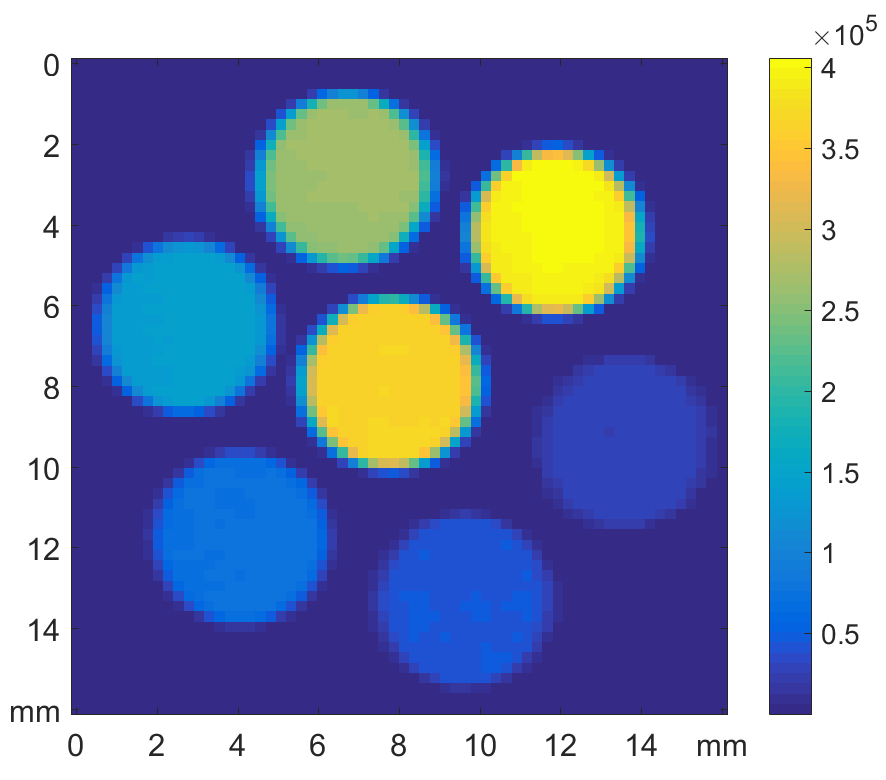

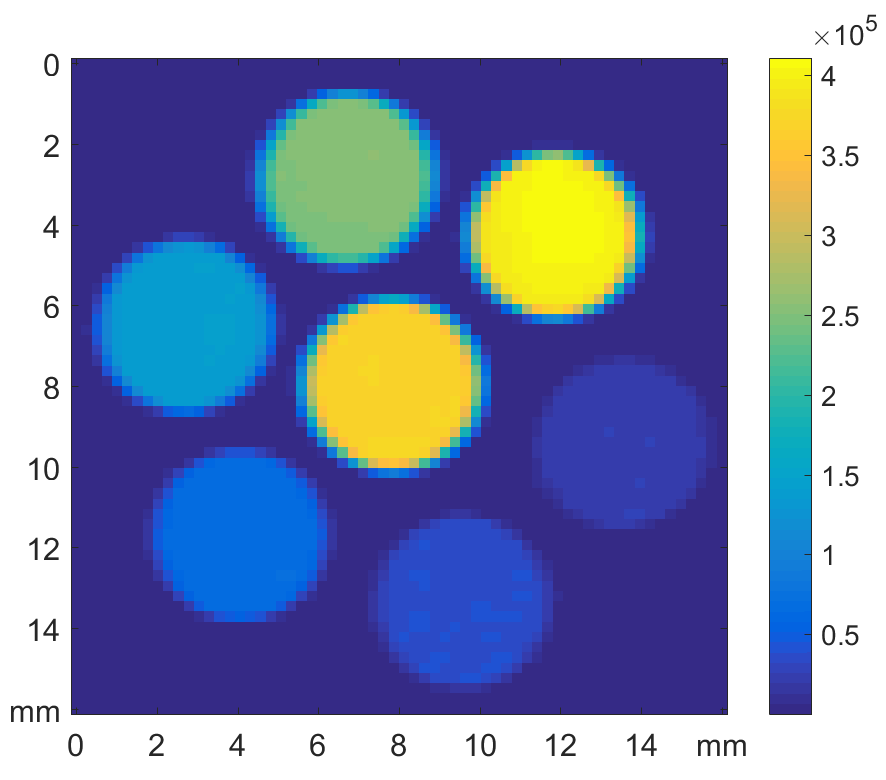

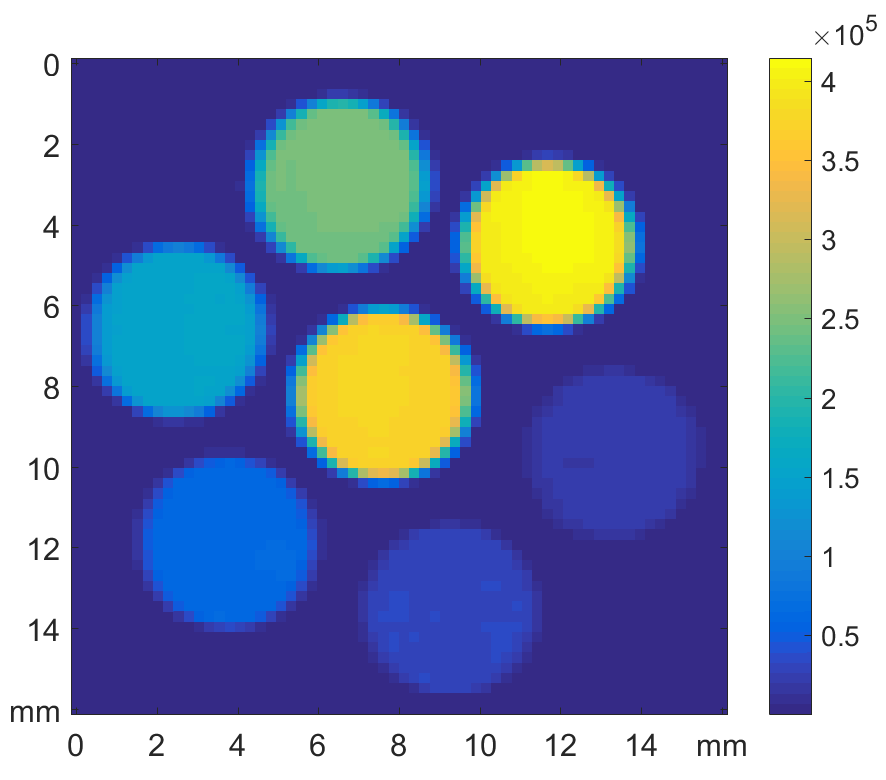

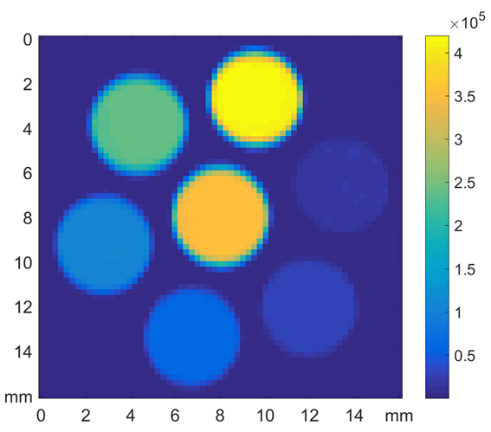


(a)

(b)

(c)

(d)

(e)

(f)


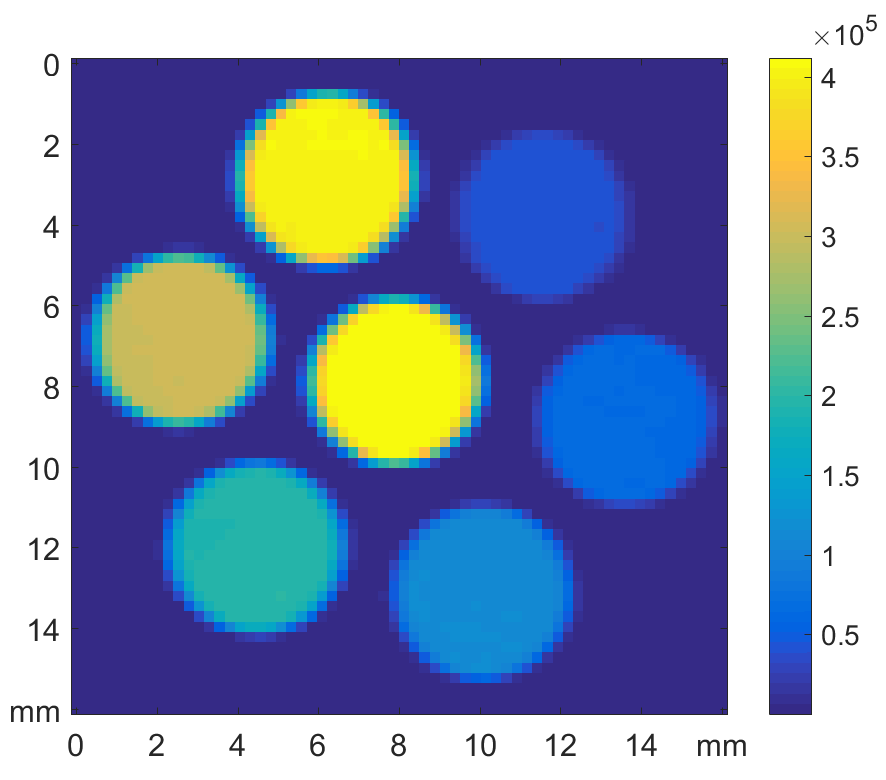

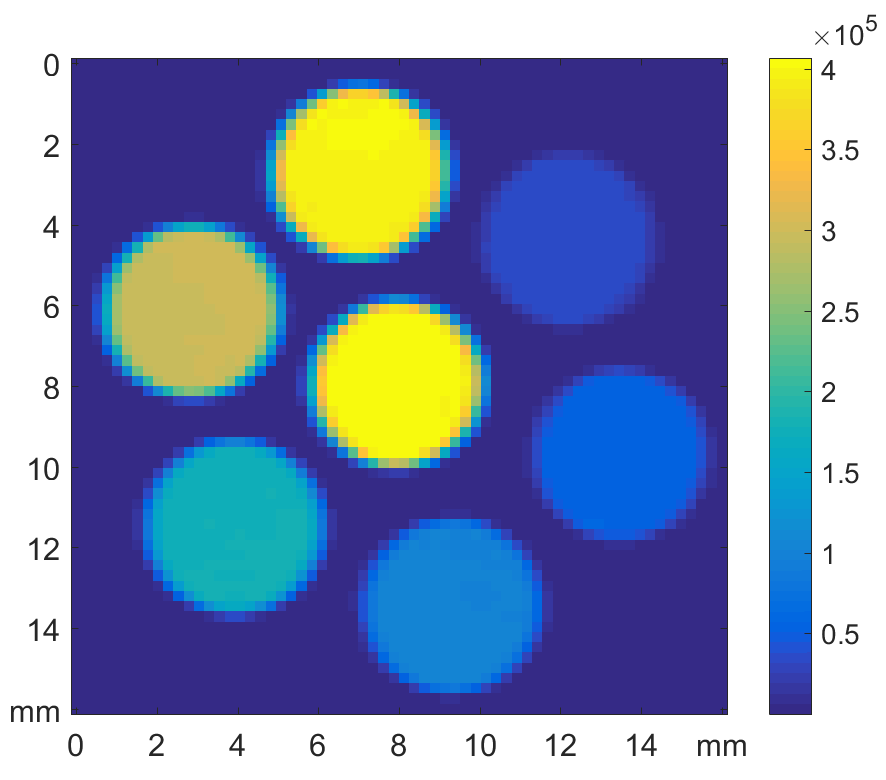

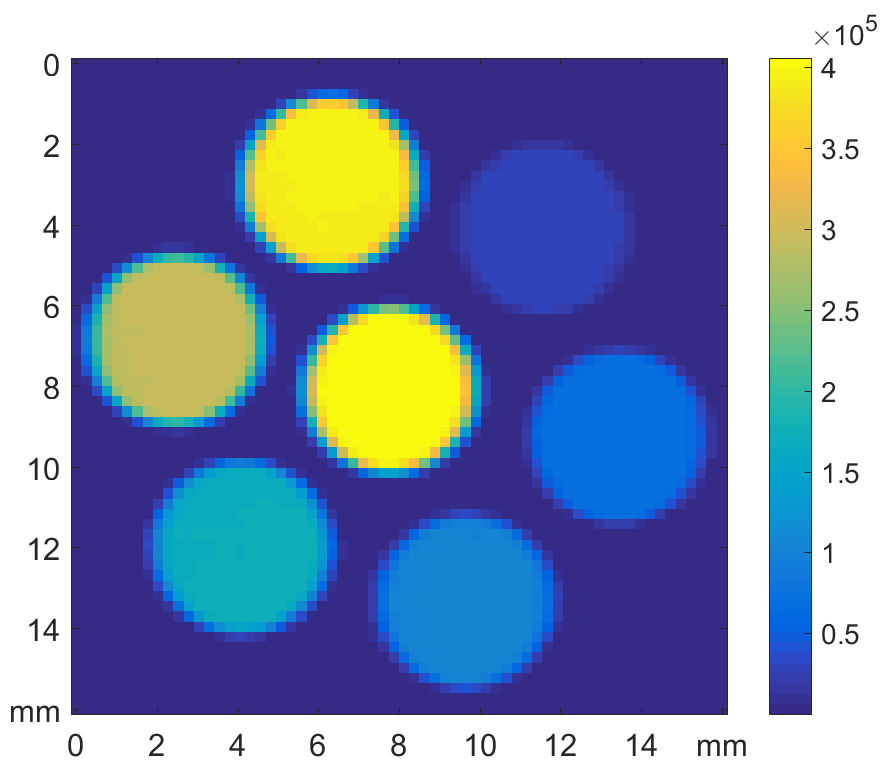

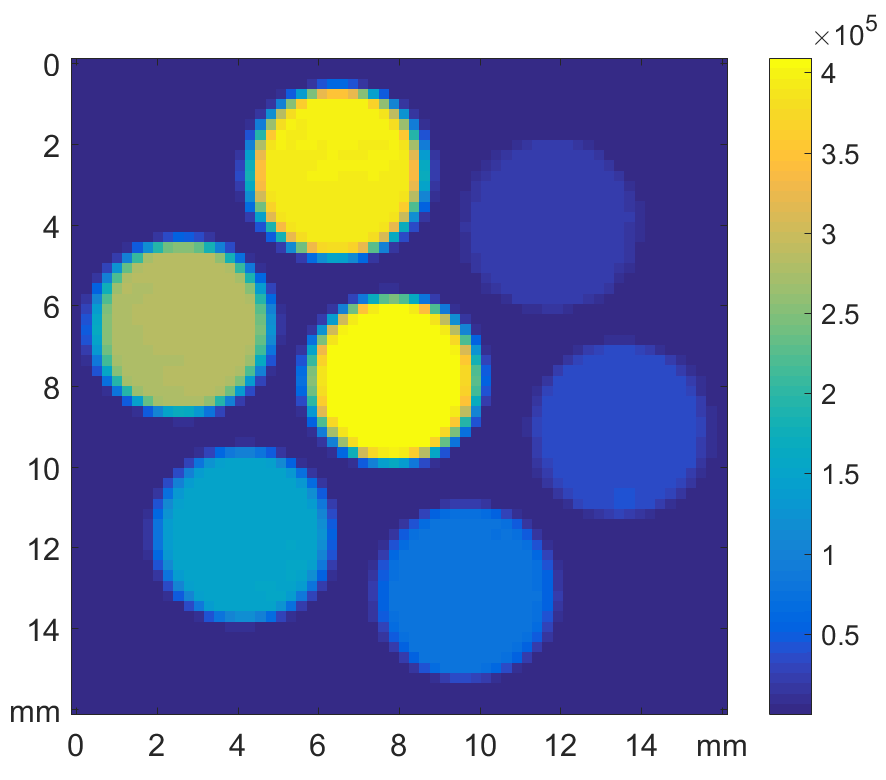

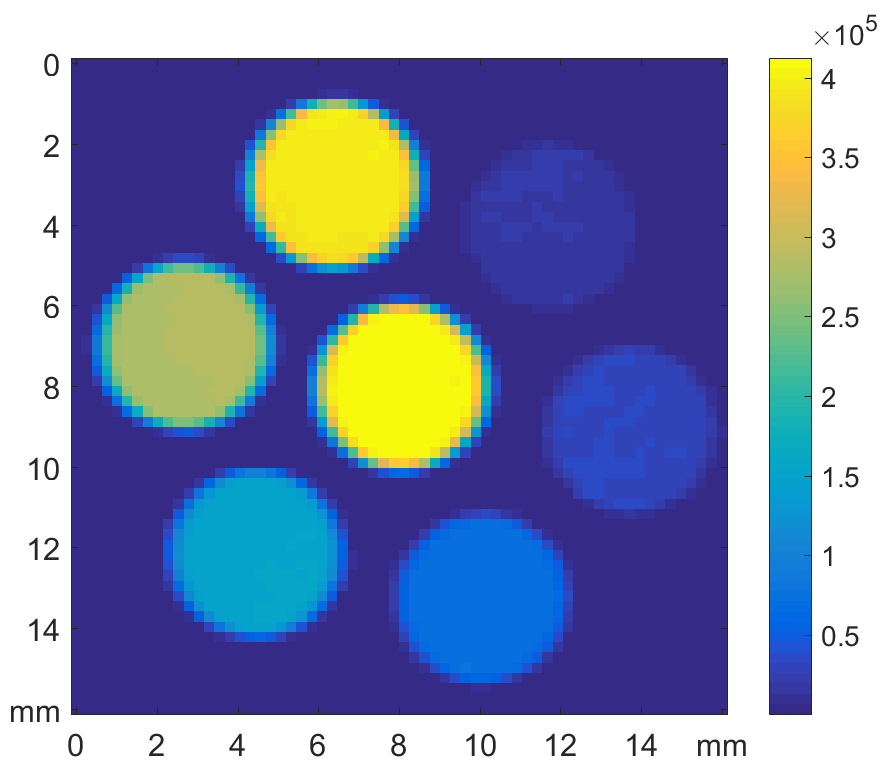

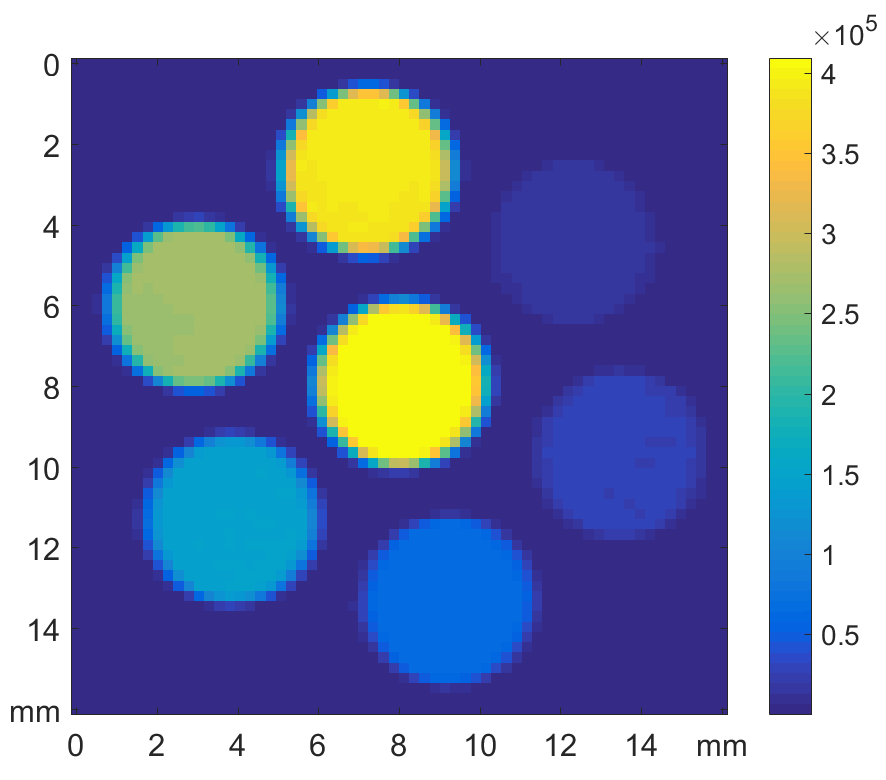


(g)

(h)

(i)

(j)

(k)

(l)

Figure S1. 1 mm thick xy-^1^H MRI RARE slice images for (a) - (f) BSA and (g) - (l) mAb calibration samples following reconstitution and mechanical swirling. Each group of seven phantom represents a serial dilution of a particular initial product concentration. Lighter (yellow) colours indicate lower BSA/mAb concentration. Each individual set of seven phantoms has been re-normalised. Serial dilution of (a) BSA 20, (b) BSA 50, (c) BSA 75, (d) BSA 100, (e) BSA 125, (f) BSA 150 , (g) mAb 20, (h) mAb 50, (i) mAb 75, (j) mAb 100, (k) mAb 125, and (l) mAb 150.


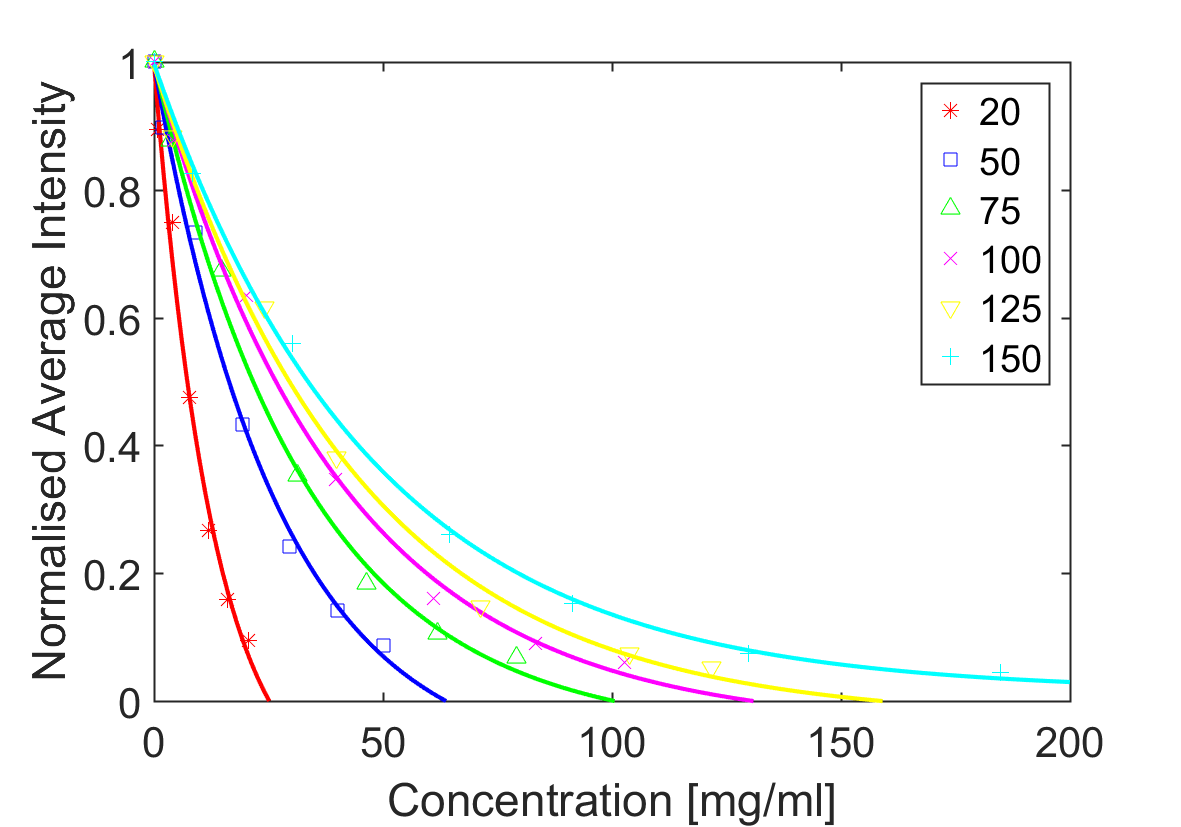


(a)

(b)


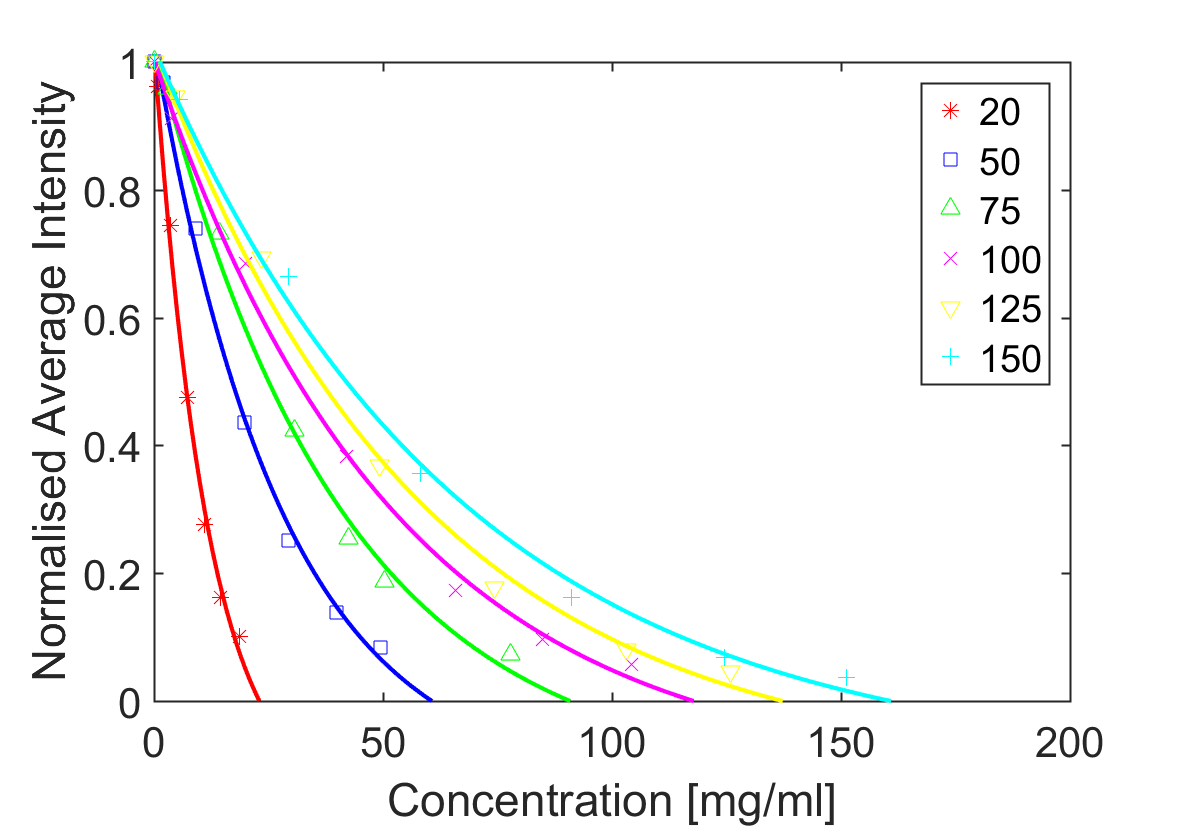


Figure S2. Normalised average ^1^H signal intensity taken using a 3 x 3 matrix of pixels from each individual phantom within a specific group of seven phantoms for each concentration. The line fitting represents the best fit using an exponential model: f(t) = aexp{-bt}+c. (a) BSA and (b) mAb samples.


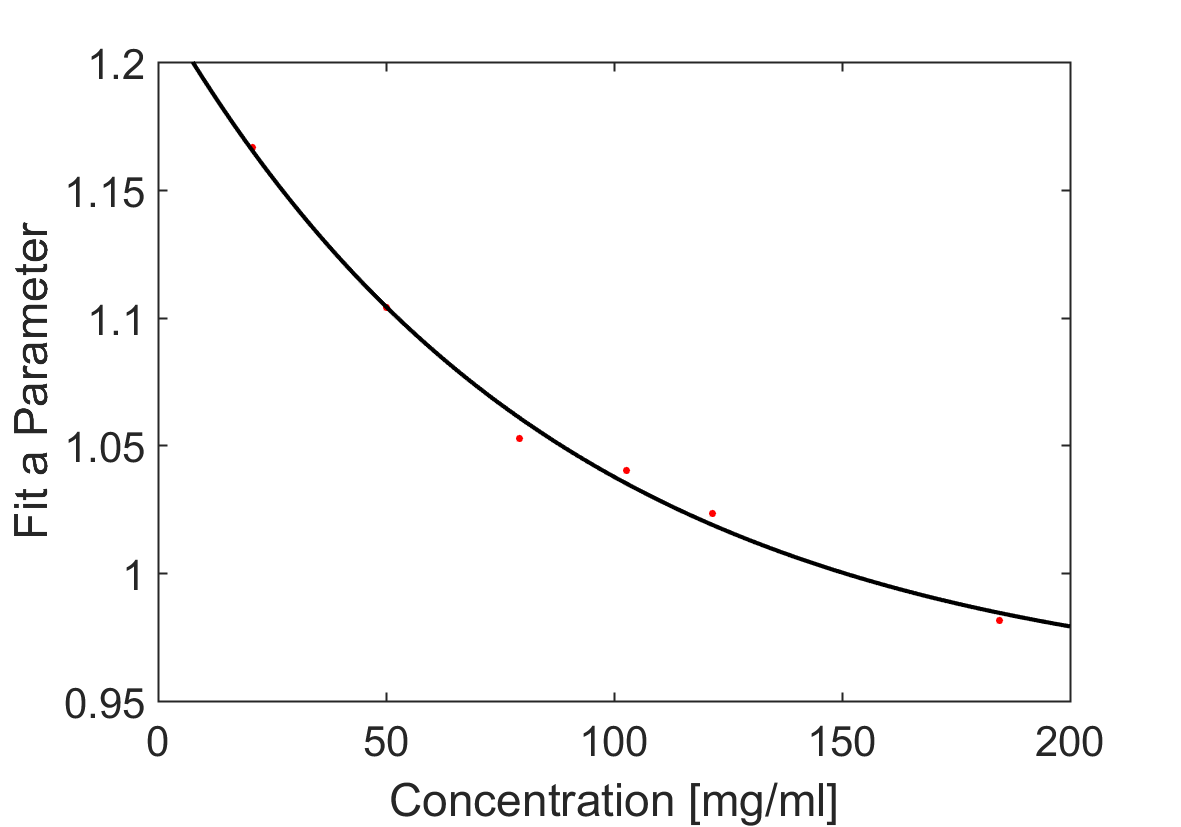

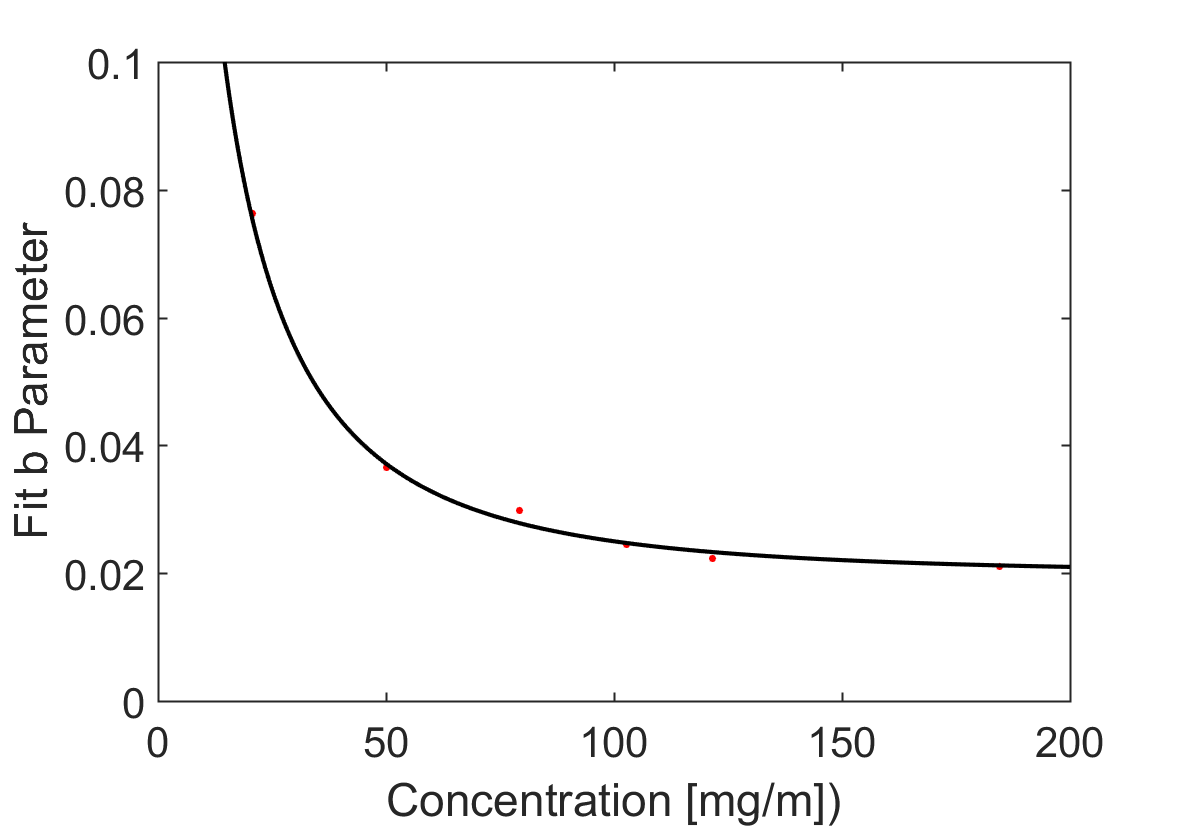

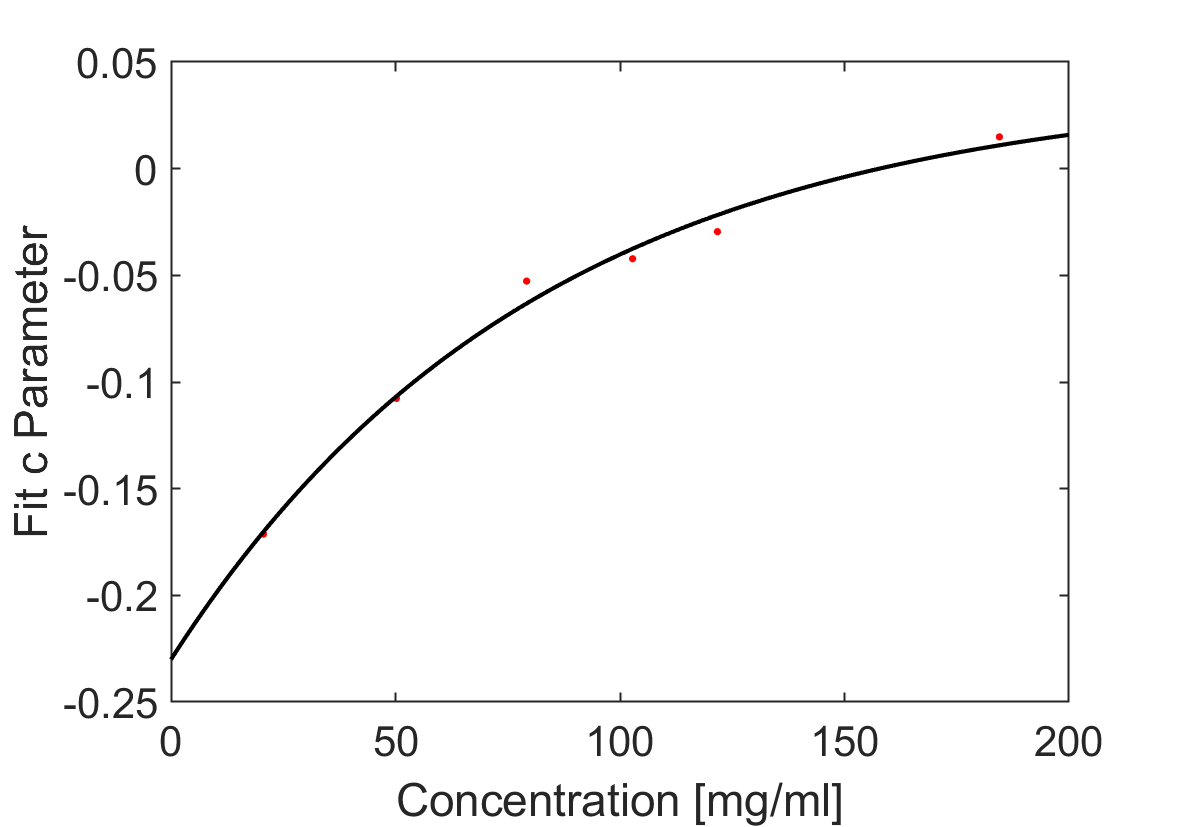


(a)

(b)

(c)


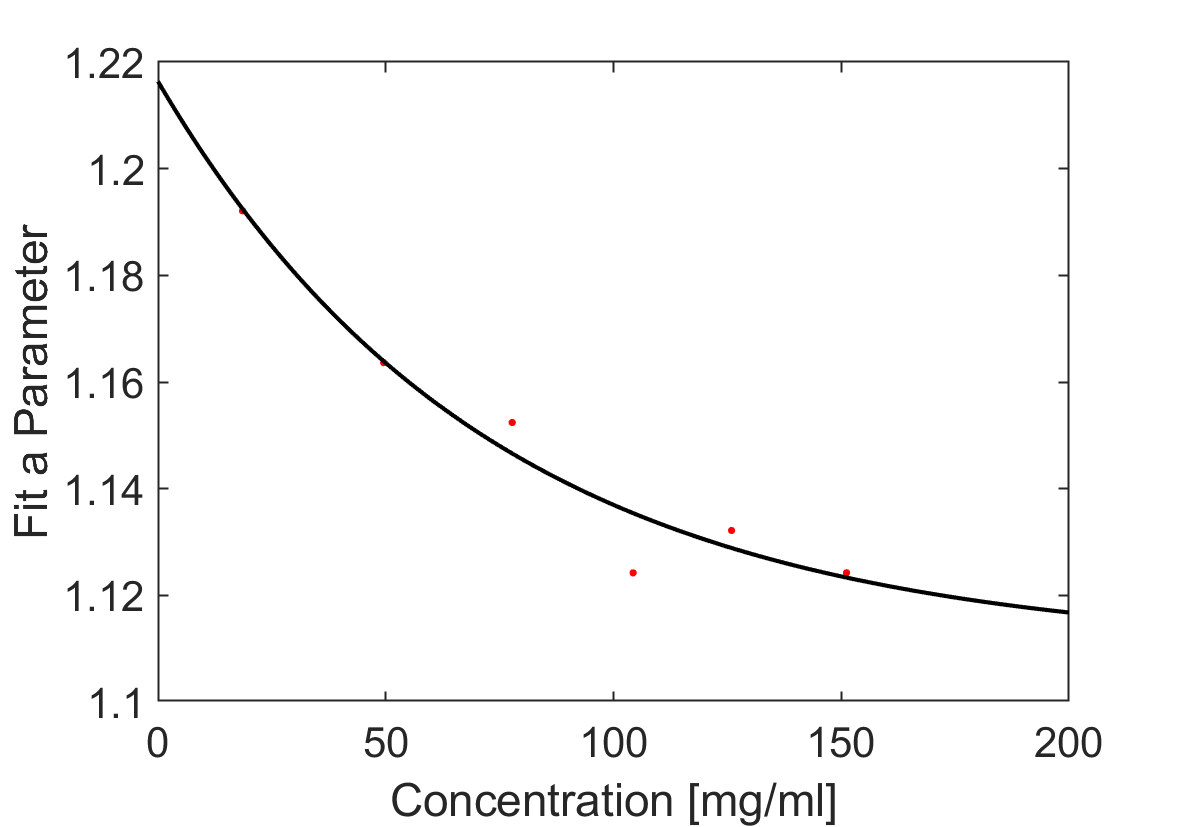

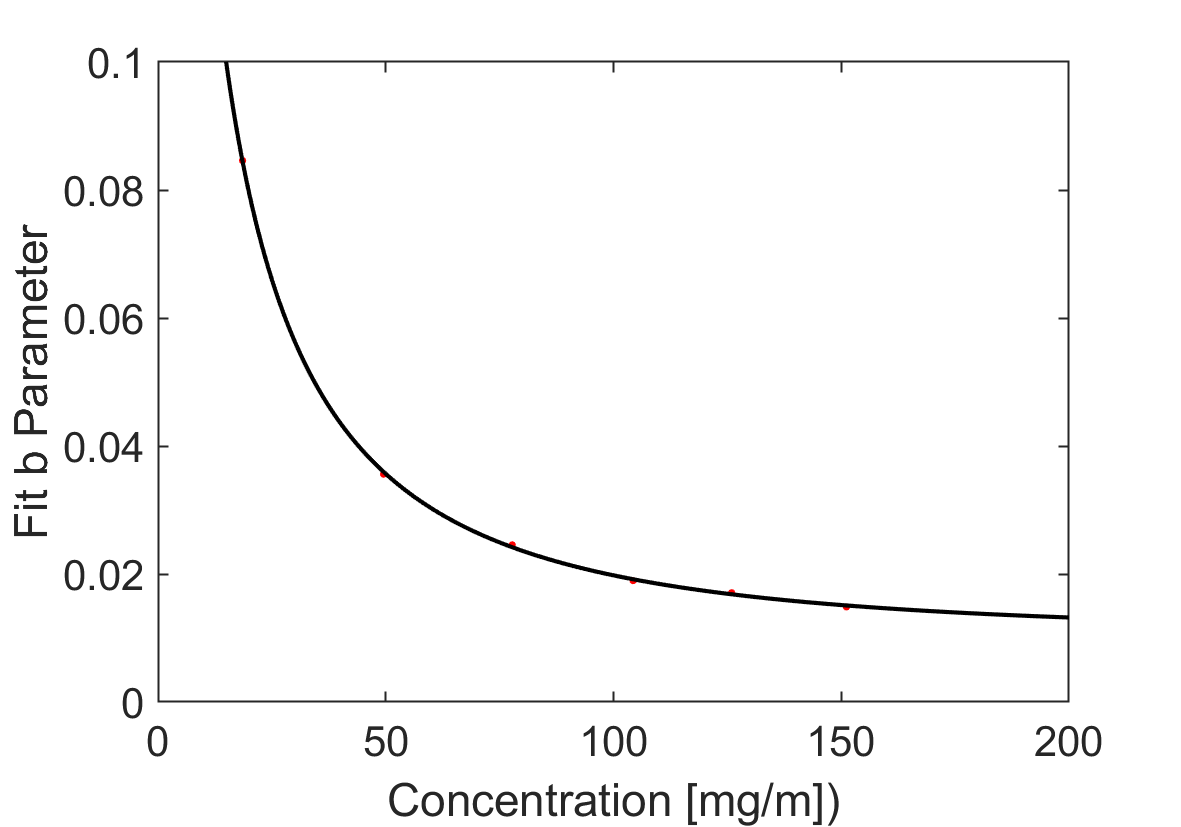

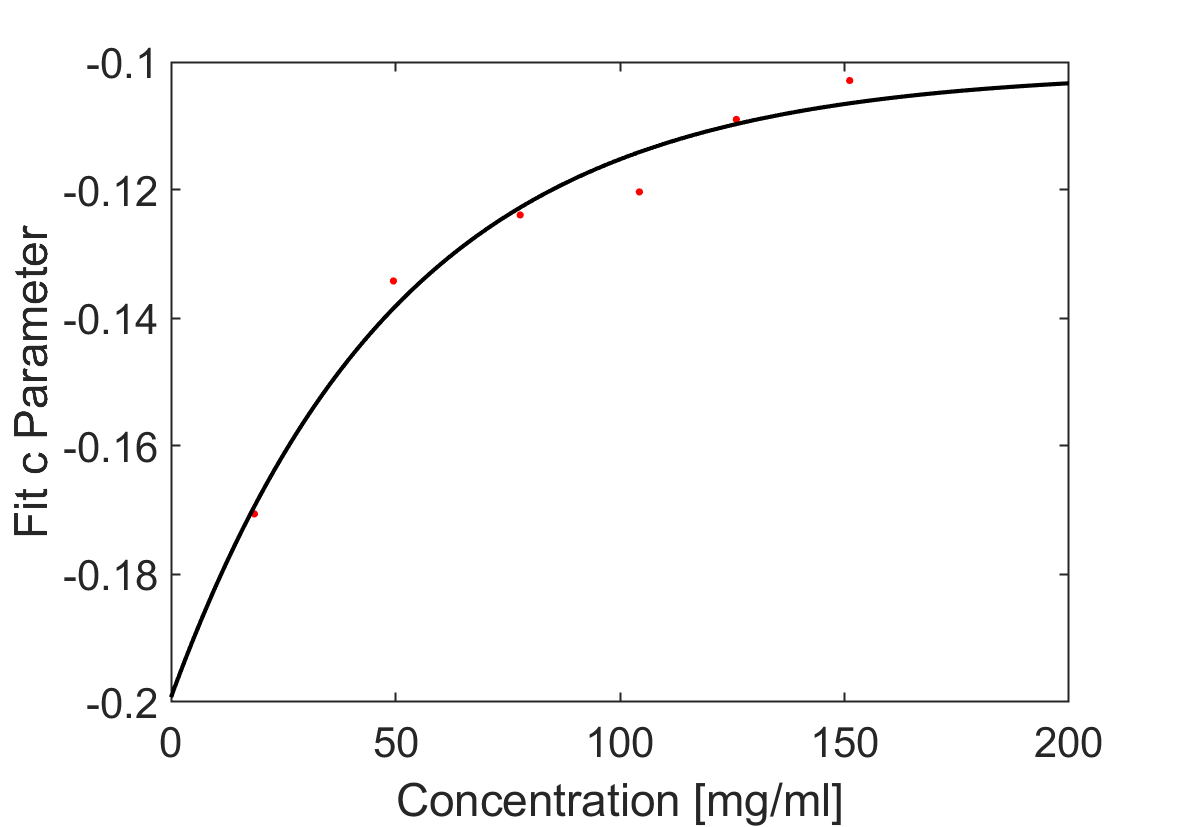


(d)

(e)

(f)

Figure S3. Parametric plots of the fitting parameters used in the exponential model, $y=aexp\left( -bx \right)+c$, described in figure S2 for BSA (a) - (c) and mAb (d) - (f). Each point represents the fit calculated from figure S2 and the lines are fits to the data for models chosen as: (a) & (d) an exponential, (b) & (e) a stretched exponential and (c) & (f) a 1 - exponential. Given a known bulk sample concentration post-swirling, the fit results calculate the correct a, b and c parameters for BSA or mAb. These can then be used with a rearrangement of $y=aexp\left( -bx \right)+c$, to calculate the concentration, *x*, given an individual (or average) pixel MRI intensity, *y*, to build a concentration map for any image acquired during reconstitution.

S4. Full reconstitution MR images available as ‘avi’ movies.

S5. Visual reconstitution videos available as ‘mp4’ files.


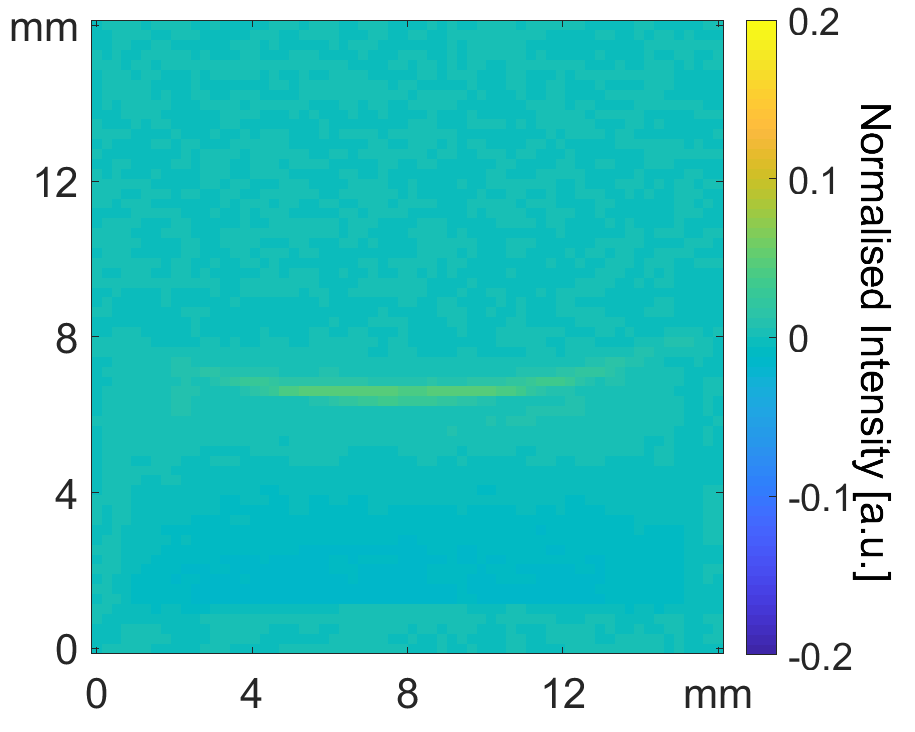

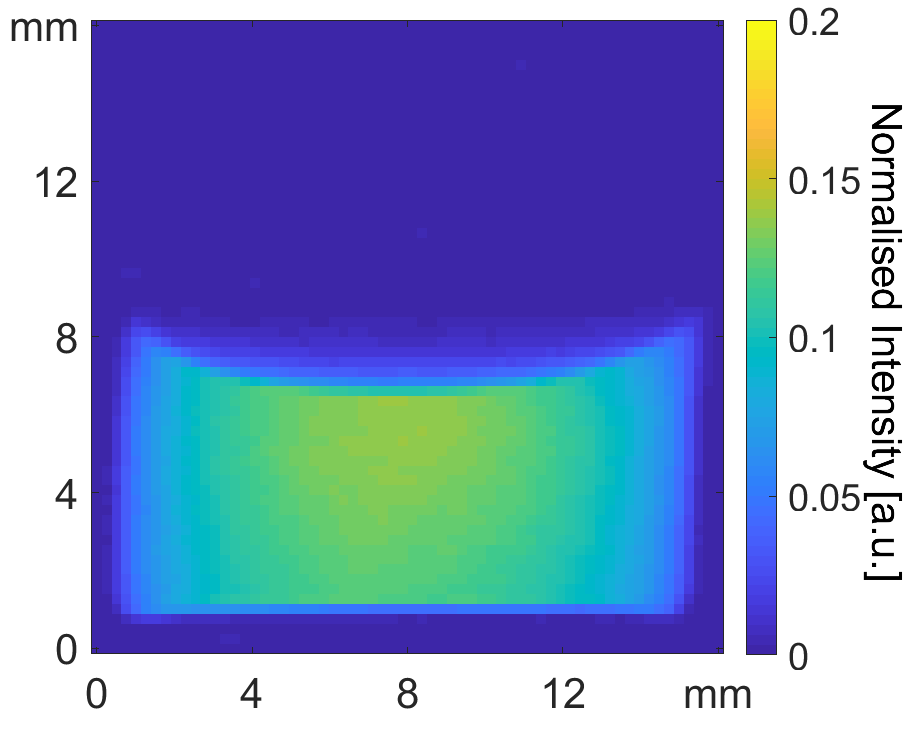

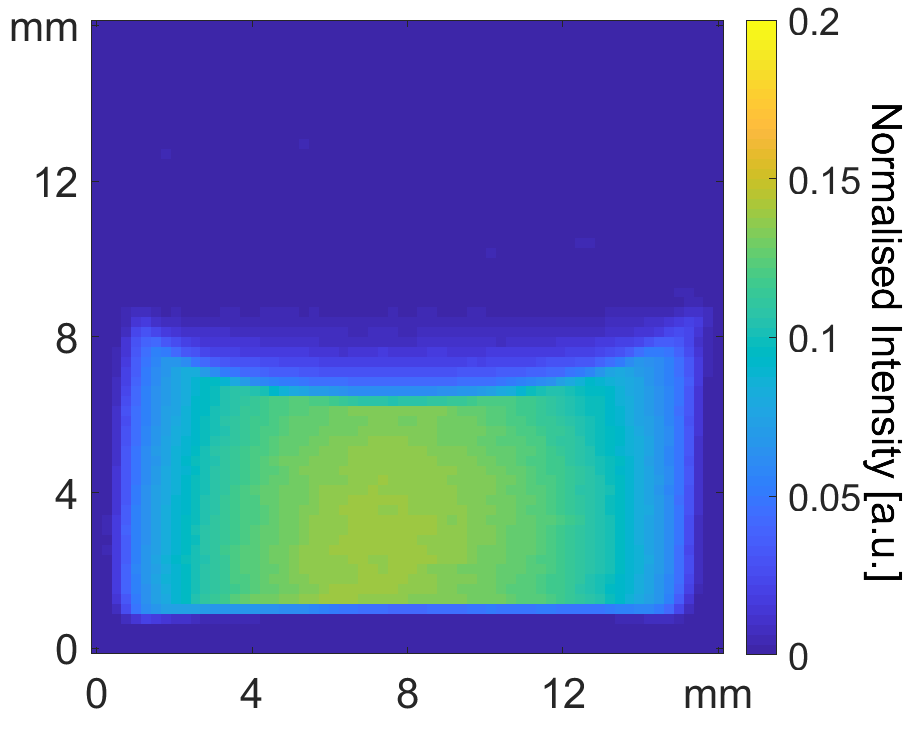


(c)

(a)

(b)


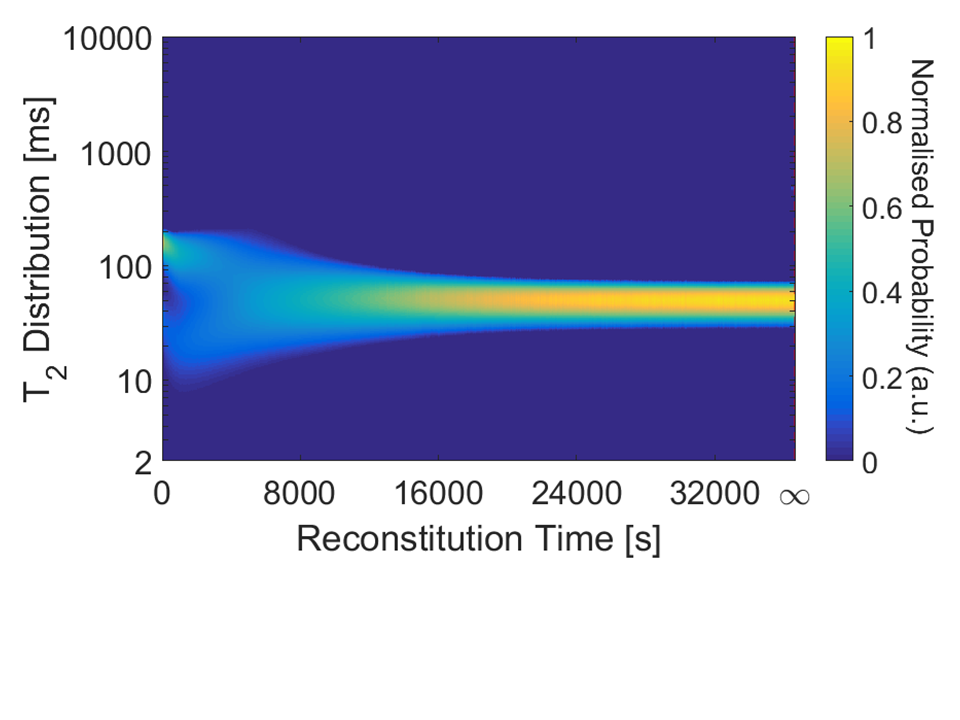


(d)

Figure S6. (a) - (c) MR Images of BSA 20 after: (a) >13 hours (48000 s) (b) Post removal and swirling, and (c) Difference of (a) - (b). The small artefacts seen in (c) are likely caused by the vial not being in exactly the same position post swirling. (d) T_2_ distribution map from one shot CPMG experiments showing the convalescence of T_2_ to a single value.


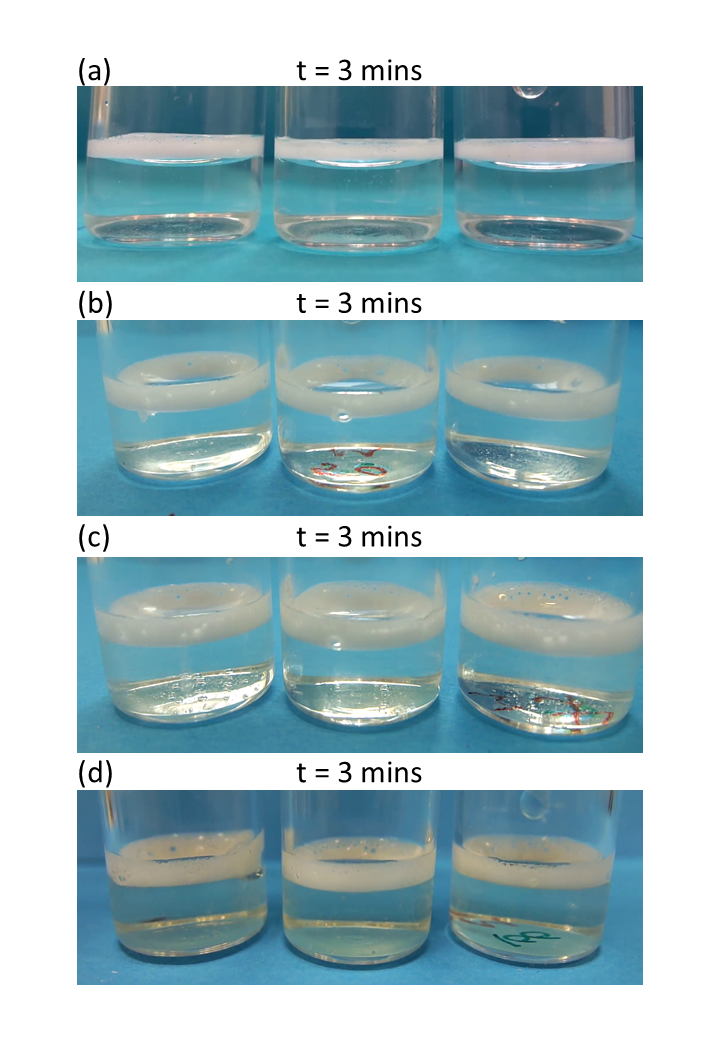


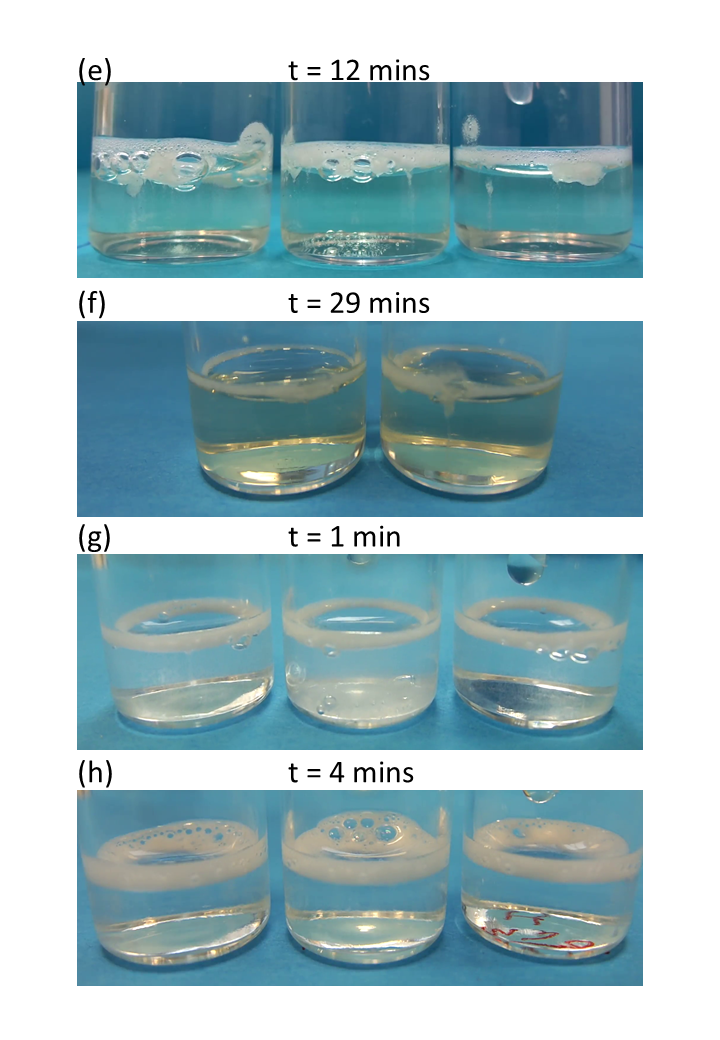


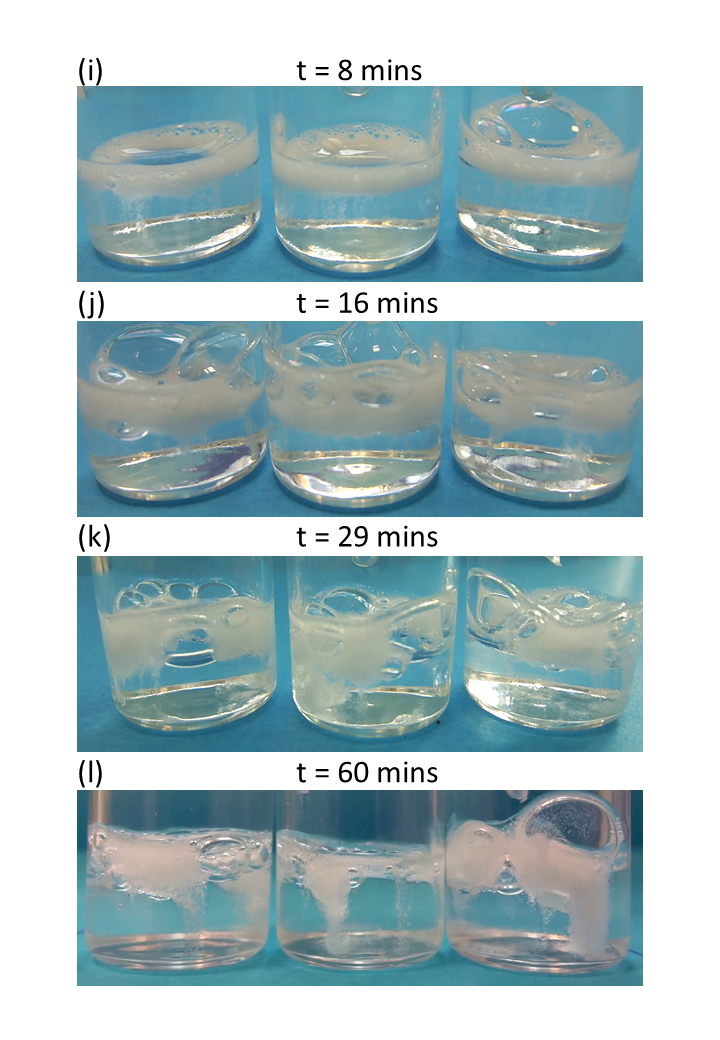


Figure S7. Snapshots from visual recons for the time points shown after injection ends for (a) BSA 20, (b) BSA 50, (c) BSA 75, (d) BSA 100, (e) BSA 125, (f) BSA 150, (g) mAb 20, (h) mAb 50, (i) mAb 75, (j) mAb 100, (k) mAb 125, and (l) mAb 150.
